# Cell-based screen identifies porphyrins as FGFR3 activity inhibitors with therapeutic potential for achondroplasia and cancer

**DOI:** 10.1172/jci.insight.171257

**Published:** 2023-11-22

**Authors:** Yun-Wen Lin, Hsiao-Jung Kao, Wei-Ting Chen, Cheng-Fu Kao, Jer-Yuarn Wu, Yuan-Tsong Chen, Yi-Ching Lee

**Affiliations:** 1 Institute of Cellular and Organismic Biology, Academia Sinica, Taipei, Taiwan.; 2 Graduate Institute of Life Sciences, National Defense Medical Center, Taipei, Taiwan.; 3 Institute of Biomedical Sciences, Academia Sinica, Taipei, Taiwan.; 4 Department of Pediatrics, Duke University Medical Center, Durham, North Carolina, USA.

**Keywords:** Bone Biology, Genetics, Bone development, Genetic diseases

## Abstract

Overactive fibroblast growth factor receptor 3 (FGFR3) signaling drives pathogenesis in a variety of cancers and a spectrum of short-limbed bone dysplasias, including the most common form of human dwarfism, achondroplasia (ACH). Targeting FGFR3 activity holds great promise as a therapeutic approach for treatment of these diseases. Here, we established a receptor/adaptor translocation assay system that can specifically monitor FGFR3 activation, and we applied it to identify FGFR3 modulators from complex natural mixtures. An FGFR3-suppressing plant extract of *Amaranthus viridis* was identified from the screen, and 2 bioactive porphyrins, pheophorbide a (Pa) and pyropheophorbide a, were sequentially isolated from the extract and functionally characterized. Further analysis showed that Pa reduced excessive FGFR3 signaling by decreasing its half-life in FGFR3-overactivated multiple myeloma cells and chondrocytes. In an ex vivo culture system, Pa alleviated defective long bone growth in humanized ACH mice (*FGFR3^ACH^* mice). Overall, our study presents an approach to discovery and validation of plant extracts or drug candidates that target FGFR3 activation. The compounds identified by this approach may have applications as therapeutics for FGFR3-associated cancers and skeletal dysplasias.

## Graphical abstract

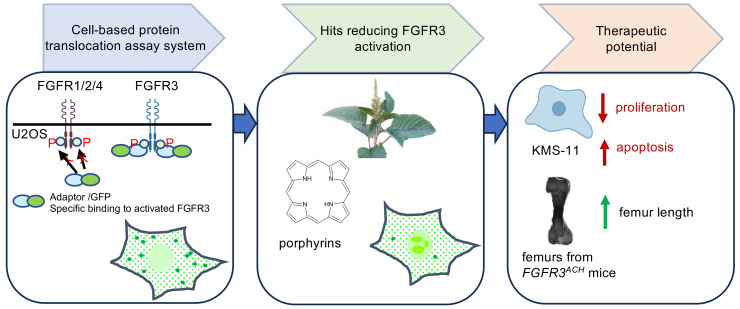

## Introduction

Fibroblast growth factor receptor 3 (FGFR3) plays an important role in regulating linear bone growth (1). Gain-of-function FGFR3 mutations have been shown to prolong FGFR3 signaling and disrupt proper chondrocyte proliferation and differentiation, leading to a group of skeletal dysplasias of varying severity (2). Among these dysplasias, achondroplasia (ACH) is the most common form of human short-limbed dwarfism, affecting more than a quarter million people worldwide. More than 99% of individuals affected with ACH possess the same FGFR3^G380R^ mutation (3–5). Meanwhile, another FGFR3-associated dysplasia, thanatophoric dysplasia type I (TDI), is more severe than ACH and is typically fatal during the perinatal period. In addition to dysplasias, abnormal FGFR3 activation is frequently found in various types of somatic cancers, and it is associated with poor prognosis or higher risk of recurrence in multiple myeloma (MM) (6), bladder carcinoma, and cervical cancer (7). Thus, FGFR3 activity is a promising therapeutic target for the treatment of various FGFR3-overactivated diseases.

Several potential therapeutic strategies have been taken to develop treatments for FGFR3 skeletal dysplasias. These include targeting the FGF ligand, FGFR3, tyrosine kinase activity of FGFR3, or FGFR3 downstream signals (8–11). Such efforts have led to the August 2021 US FDA approval of the first treatment for patients with ACH, a C-type natriuretic peptide analog called vosoritide (Voxzogo), which partially inhibits FGFR3 downstream signaling (12). However, the strategy of suppressing certain FGFR3 downstream pathways is insufficient to prevent multiple intracellular signals initiated by FGFR3. Moreover, current strategies block the activity of FGFR3 with low specificity, especially tyrosine kinase inhibitors, which compete with the ATP binding site of receptor tyrosine kinases (13, 14). Thus, the identification and development of small molecules that directly target FGFR3 activity with high specificity could yield much-needed new therapeutic candidates.

As natural products have extensive structural diversity and screens of plant extracts often produce hits with high specificity toward biological targets (15), we focused on identifying natural molecules that specifically inhibit FGFR3 at early activation stages. However, the discovery of bioactive compounds from complex mixtures is challenging, as the approach involves steps of screening, isolation, and functional characterization (16). Here, we developed and validated a cell-based system that can be used to specifically monitor early FGFR3 activation and allowed us to screen for FGFR3 modulators from complex mixtures of plant extracts. By doing so, we identified a potential therapeutic compound, pheophorbide a (Pa), and demonstrated that it effectively suppresses FGFR3 activity by reducing FGFR3 half-life and its downstream signaling in both FGFR3-overactivated MM cells and chondrocytes. Pa efficacy was further validated in FGFR3-overactivated MM cells, where it reduced cell proliferation and induced cell apoptosis. Using our previously established humanized ACH mouse model (*FGFR3^ACH^* mice) (17), we further demonstrated that Pa is capable of alleviating defective growth of long bones. In sum, this study introduces a reliable platform for screening potential therapeutic components from plant extracts to target FGFR3-mediated diseases, which may be useful leads for the development of treatments.

## Results

### A cell-based receptor/adaptor translocation assay to specifically monitor FGFR3 activity.

**Figure 1:**
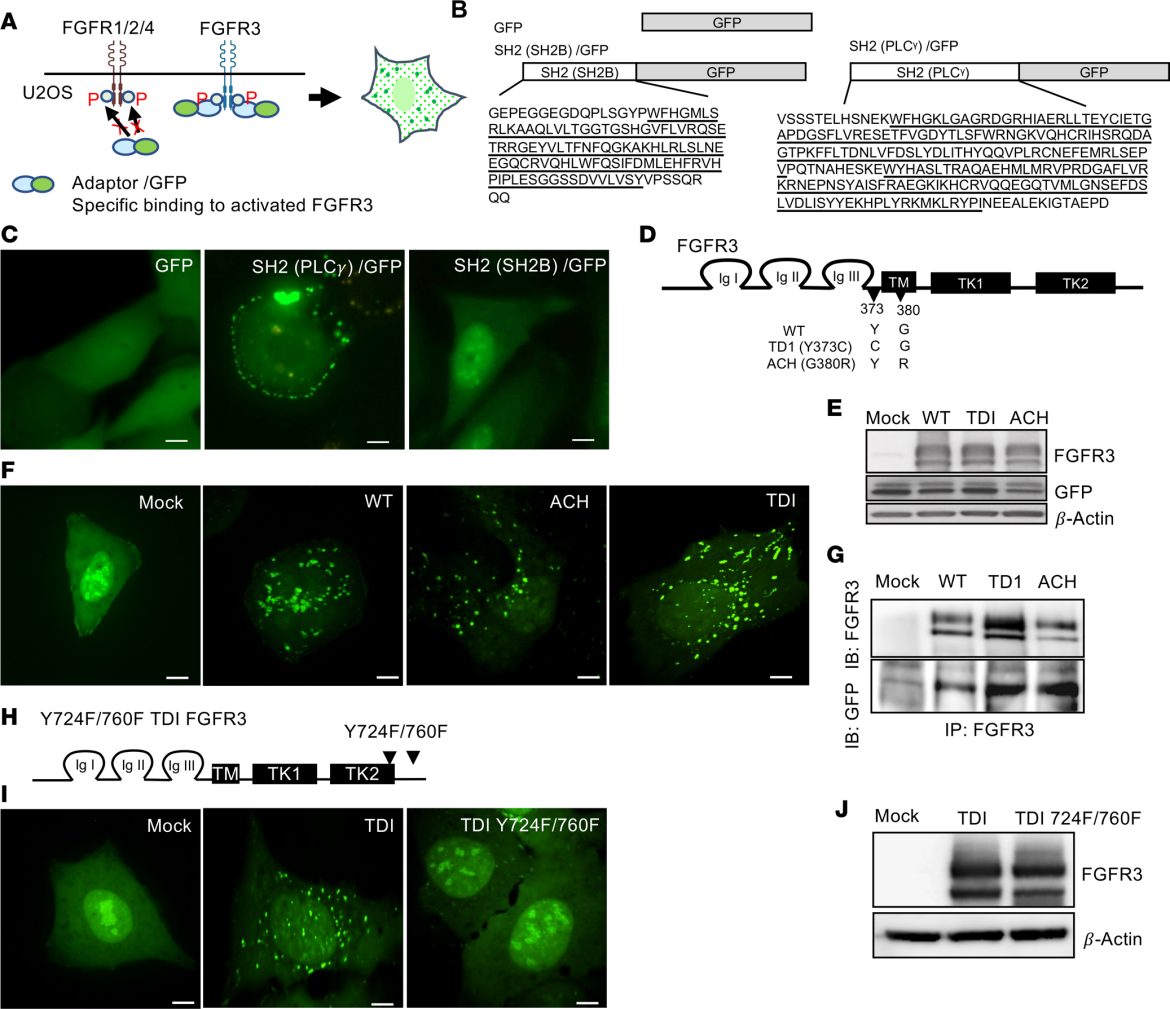
SH2 (SH2Bβ)/GFP specifically interacts with activated FGFR3 to generate an internalized cytoplasmic spot pattern in U2OS cells. (**A**) Schematic illustration shows the assay design concept by tracking the subcellular localization of an adaptor/GFP fusion protein that specifically interacts and internalizes with activated FGFR3. (**B**) Schematic representation shows the SH2 domain (underlined) of SH2Bβ and PLCγ proteins fused to the N-terminus of GFP. (**C**) U2OS cells transiently expressing GFP, SH2(SH2B)/GFP, or SH2(PLCγ)/GFP were observed by confocal live imaging. Scale bars, 10 μm. (**D**) Schematic representation shows the relative positions of different mutations in FGFR3 known to cause skeletal dysplasias. TDI, thanatophoric dysplasia 1; Ig I, II, and III; immunoglobulin-like domains; TM, transmembrane I domain; TK1 and 2, tyrosine kinase domains. (**E**) U2OS cells stably coexpress SH2 (SH2B)/GFP and various FGFR3s (WT, ACH, and TDI) or empty vector control (mock). The levels of FGFR3 and SH2 (SH2B)/GFP fusion proteins were detected by immunoblotting. (**F**) SH2 (SH2B)/GFP fluorescence signals (green) in the indicated cell lines were observed by confocal live imaging. Scale bars, 10 μm. (**G**) The direct interaction of SH2(SH2B)/GFP and FGFR3 was detected by co-immunoprecipitation (IP) using FGFR3 antibody followed by immunoblotting for FGFR3 and GFP. (**H**–**J**) Inhibition of FGFR3 activation by mutated Y724 and Y760 phosphorylation sites in TDI FGFR3 abolished the internalized spot pattern in U2OS cells coexpressing SH2(SH2B)/GFP cells TDI FGFR3. (**H**) Schematic representation shows the substitution of phenylalanine for tyrosine at Y724F and Y760F in TDI-FGFR3. (**I**) U2OS-SH2(SH2B)/GFP cells stably expressing empty vector control (mock), TDI-FGFR3, or Y724F/Y760F TDI-FGFR3 were observed by confocal live imaging. Scale bars, 10 μm. (**J**) The expression of FGFR3 was detected by immunoblotting.

Plant extracts have long been recognized as a valuable source of bioactive molecules for pharmacotherapy and drug development (16). In this study, our focus was to identify natural compounds from plant extracts that specifically inhibit FGFR3 at early activation stages. To achieve this aim, we established a cell screening system capable of specifically monitoring early FGFR3 activation, while minimizing interference from autofluorescence or fluorescence-quenching compounds present in plant extracts. The activation of FGFR3 initiates a cascade of events, including receptor dimerization, tyrosine phosphorylation in the receptor kinase domain, adaptor protein recruitment, and internalization of the activated FGFR3-adaptor protein complex (3). We designed a system to monitor early FGFR3 activation by tracking the subcellular localization of an adaptor/GFP fusion protein that specifically interacts and internalizes with activated FGFR3. The schematic representation of the whole-cell FGFR3 assay system is depicted in Figure 1A. Notably, cells ectopically expressing either wild-type (WT) or activated mutant FGFR3, together with adaptor/GFP proteins that selectively bind to activated FGFR3, are expected to exhibit intracellular punctate fluorescent signals. These signals indicate the presence of internalized vesicles containing activated FGFR3 and adaptor/GFP complexes. By tracking changes in the subcellular localization of an FGFR3-specific interacting adaptor/GFP, we aimed to identify natural compounds from plant extracts that can inhibit FGFR3 at an early activation stage.

To establish the system, our first objective was to identify an adaptor protein that specifically interacts with activated FGFR3. We elected to use U2OS cells as a model to identify the desired adaptor protein, as we found this human osteosarcoma cell line endogenously expressed FGFR1, 2, and 4 but had no detectable FGFR3 expression (Supplemental Figure 1; supplemental material available online with this article; https://doi.org/10.1172/jci.insight.171257DS1). To ensure minimal interference from downstream FGFR3 signaling on cell proliferation or viability, we utilized only the binding domain of the adaptor protein rather than the full-length sequence. We investigated the Src homology 2 (SH2) domain of 2 known adaptor proteins that interact with FGFRs or FGFR3 in U2OS cells. The SH2 domain of PLCγ binds to tyrosine-autophosphorylated regions of FGFRs (18), while the SH2 domain of SH2Bβ was identified through a yeast 2-hybrid screen for its interaction with activated kinase domains of FGFR3 (19). To visually examine the intracellular localization and interactions of these SH2 domains with endogenous proteins in U2OS cells, we fused them to GFP (Figure 1B). When U2OS cells expressed SH2(PLCγ)/GFP, we observed punctate signals at the plasma membrane, indicating its interaction with endogenous plasma membrane proteins. In contrast, U2OS cells expressing SH2(SH2B)/GFP exhibited a diffuse fluorescent signal throughout the cytoplasm and nucleus, accompanied by few condensed fluorescent signals in nucleus (Figure 1C). Of note, SH2Bβ is known to undergo nucleocytoplasmic shuttling and has functions in nucleus (20). In the absence of an interaction between SH2(SH2B) and endogenously expressed membrane proteins in U2OS cells, the SH2(SH2B) may interact with proteins located in nucleus, leading to the observed condensed signals within the nucleus. These findings demonstrate that SH2(SH2B)/GFP does not interact with endogenously expressed plasma membrane proteins in U2OS cells, including FGFR1, 2, and 4.

To explore the interaction between FGFR3 and SH2(SH2B)/GFP in U2OS cells, we generated stable cell lines expressing SH2(SH2B)/GFP along with WT or activating mutation forms of FGFR3 (Figure 1D). Western blot analysis verified the expression of SH2(SH2B)/GFP and WT or mutant FGFR3 in U2OS cells (Figure 1E). Notably, U2OS cells coexpressing both SH2(SH2B)/GFP and different forms of FGFR3 (WT, ACH, and TDI) displayed distinct intracellular punctate GFP signals (Figure 1F). These cells also displayed fewer nuclear condensed signals than control cells (mock) that only expressed SH2(SH2B)/GFP (Figure 1F). Additionally, weak plasma membrane signals were observed in U2OS cells coexpressing SH2(SH2B)/GFP and the different forms of FGFR3 (WT, ACH, and TDI) (Figure 1F). These intracellular punctate signals suggest that the recruitment of SH2(SH2B)/GFP with activated FGFR3 (WT, ACH, and TDI) lead to internalization and formation of intracellular puncta. Furthermore, the direct interaction between FGFR3 and SH2(SH2B)/GFP was validated through co-immunoprecipitation of SH2(SH2B)/GFP with FGFR3 variants (Figure 1G).

To directly investigate the relationship between FGFR3 activation and intracellular punctate GFP signal, we examined the impact of inhibiting FGFR3 activity. We inactivated a constitutively active TDI-FGFR3 by substituting 2 critical tyrosine (Y) phosphorylation sites with phenylalanine (F) residues, Y724F and Y760F (Figure 1H). These 2 phosphorylation sites are well established as vital for FGFR3 activation and its interaction with the SH2 domain of SH2B (19). Strikingly, the intracellular punctate GFP signals were completely abolished in U2OS cells coexpressing SH2(SH2B)/GFP and Y724F/Y760F TDI-FGFR3 (Figure 1I), while the expression levels of both FGFR3 isoforms were similar between the groups (Figure 1J).

These findings highlight the successful establishment of a cell-based receptor/adaptor translocation system for specifically monitoring FGFR3 activity. By utilizing SH2(SH2B)/GFP, we demonstrated the direct interaction between SH2(SH2B)/GFP and activated FGFR3, leading to intracellular punctate GFP signals in U2OS cells.

### High-throughput imaging assay to quantify FGFR3 activity.

Our next goal was to establish a high-throughput imaging method to quantify FGFR3 activity for drug screening. The TDI Y373C substitution in the extracellular region of FGFR3 (TDI-FGFR3) results in the formation of intermolecular disulfide bonds that cause receptor dimerization, constitutive kinase activation, and adaptor protein recruitment (2). Since U2OS cells expressing SH2(SH2B)/GFP and TDI-FGFR3 (U2OS-TDI/SH2GFP) exhibit robust intracellular GFP puncta, we used this cell line to establish high-throughput imaging system (Figure 1). With this system, we are able to screen for hits that inhibit early events of FGFR3 activation, including disrupting receptor dimerization, kinase activation, and adaptor protein recruitment.

**Figure 2:**
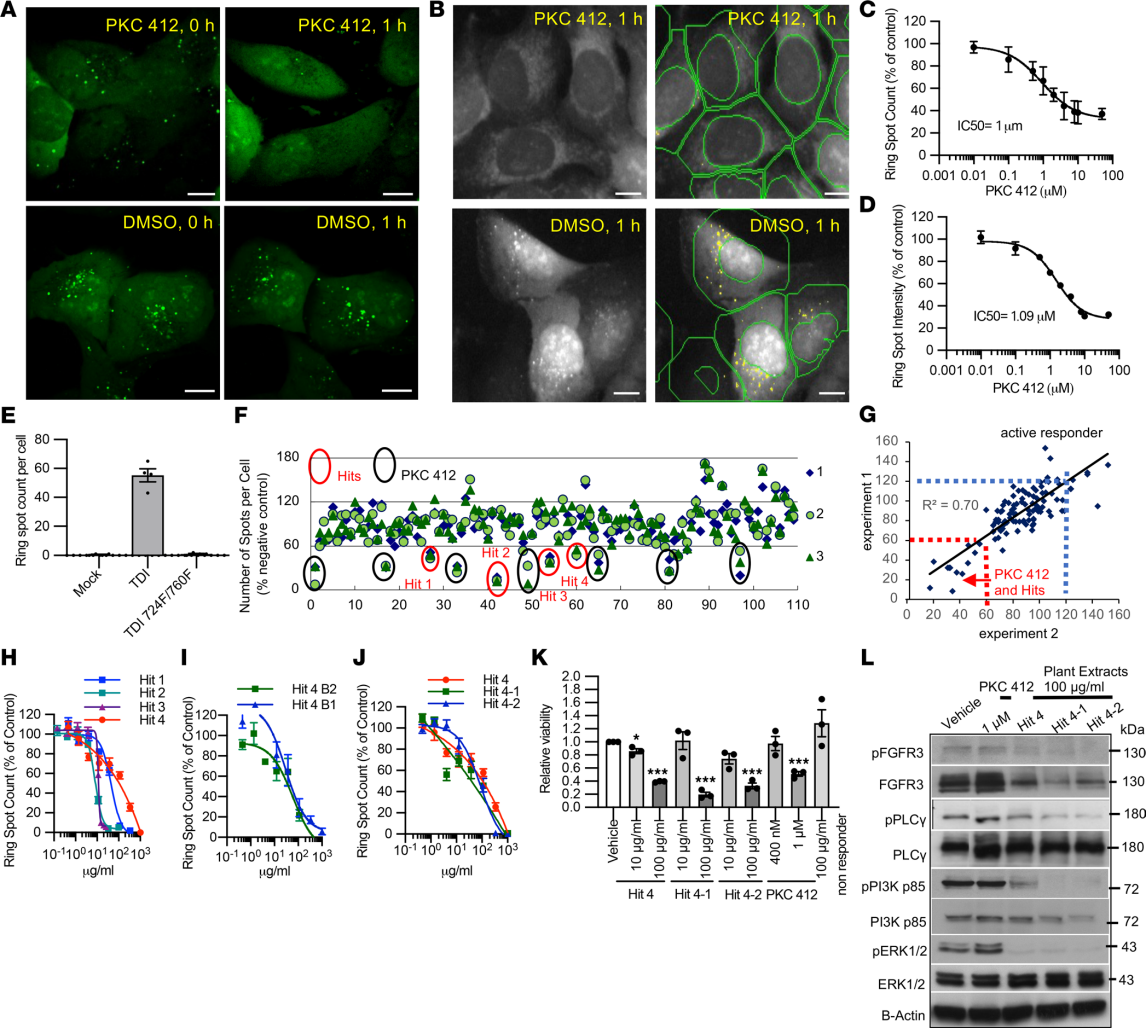
High-throughput imaging system (receptor/adaptor translocation assay) to quantify FGFR3 activation and identify hits that inhibit FGFR3 activation. (**A** and **B**) U2OS-TDI/SH2BGFP cells were treated with 4 μM PKC412 or vehicle control (DMSO) for 1 hour. Scale bars, 20 μm. (**A**) Confocal images show SH2(SH2B)/GFP signals (green). (**B**) Representative raw images acquired on the ArrayScan VTI HCS Reader (left panels) and automated identification of spots (yellow dots) within cytoplasmic area (green ring) (right panels). (**C** and **D**) Dose-response curves of PKC412 plotted by ring spot count (**C**) or ring spot intensity (**D**) relative to vehicle control. (**E**) FGFR3 activity was quantified based on ring spot counts in U2OS-SH2(SH2B)/GFP cells expressing TDI FGFR3, Y724F/760F TDI FGFR3, or vector control (Mock). (**F**) U2OS-TDI/SH2BGFP cells were treated with different plant extracts. Ring spot count per cell was plotted relative to vehicle control. Data from 3 independent treatments are shown. Hits: red circles; PKC412: black circles. (**G**) Linear regression analysis of experiments 1 and 2 showing PKC412 treatments and hits (lower-left corner) and active responder (upper-right corner). (**H**–**J**) Dose-response curves of (**H**) 4 identified hits (Hits 1–4), (**I**) 2 different batches of Hit 4 (Hit 4 B1 and Hit 4 B2), and (**J**) 2 additional plant extracts from species closely related to Hit 4: Hit 4-1 (*A*. *viridis*) and Hit 4-2 (*A*. *tricolor*). Data in **C**–**E** and **H**–**J** represent the mean ± SEM of triplicates. (**K**) KMS-11 cells were treated with vehicle control or different doses of plant extracts for 48 hours. Cell viability was analyzed using WST-1 assay and normalized to the vehicle control. Data represent the mean ± SEM of 3 independent experiments. Student’s 2-tailed t tests were performed. *P < 0.05, ***P < 0.001. (**L**) KMS-11 cells were treated with indicated concentrations of plant extracts, PKC412, or vehicle control (Vehicle) for 1 hour. Total and phosphorylated protein levels of FGFR3 and its downstream effectors were detected by immunoblotting.

To optimize the protocol and parameter settings for the high-throughput imaging system, we used PKC412, a small-molecule multiple tyrosine kinase inhibitor, which has been shown to inhibit FGFR3 tyrosine phosphorylation (21). Indeed, treating U2OS-TDI/SH2GFP cells with PKC412 for 1 hour led to a marked reduction in intracellular punctate GFP signals (Figure 2A). To automatically quantify the punctate GFP signals in cytoplasm, we employed nuclei staining using Hoechst to identify individual cells, and cell images were automatically obtained via the ArrayScan VTI HCS Reader. For each cell, the nucleus (circle) and cytoplasmic area (ring) were defined using the parameter settings in the Compartmental Analysis BioApplication software (Figure 2B). The GFP spot number (ring spot count) and spot intensity (ring spot intensity) in ring area were determined.

To evaluate the principal quantifiable features reflecting the underlying biology, we conducted a dose-response assay with PKC412 and plotted the ring spot counts and ring spot intensity per cell as percentage of DMSO control (Figure 2, C and D). Both parameters reliably showed a dose-dependent response to the inhibitor, indicating their sensitivity to FGFR3 activation. However, considering the potential influence of autofluorescence or fluorescence-quenching compounds present in plant extracts on the ring spot intensity, we decided to use the ring spot count per cell as the preferred method for routine quantification of FGFR3 activation.

To verify the capability of the system to quantify FGFR3 inactivation, we used it to compare U2OS-TDI/SH2GFP cells with U2OS-Y724F/760F TDI/SH2GFP cells. The dramatic reduction in the number of intracellular spot counts in U2OS-Y724F/Y760F TDI/SH2GFP cells (Figure 2E) supported the notion that the counts of intracellular spots directly reflect the degree of FGFR3 activation. Therefore, we utilized this high-throughput FGFR3-TDI/SH2GFP imaging system, which we termed R/ATA (receptor/adaptor translocation assay), for subsequent experiments.

### Identification of Amaranthus viridis extract as an inhibitor of FGFR3 activation.

The R/ATA system was used to screen a library of plant extracts from various species collected in Taiwan, aiming to identify hits that could effectively reduce FGFR3 activation. To identify hits targeting the early events of FGFR3 activation, we treated cells with the plant extracts for 1 hour prior to analysis. A hit was defined as a treatment that exhibited more than 40% inhibition of FGFR3 activity in 3 independent experiments. This criterion was established based on the observation that the control inhibitor (PKC412) suppressed at least 40% of FGFR3 activity in our experiments. Using the 40% inhibition criterion, we identified 4 plant extracts as hits from the initial screen (Figure 2F). The inhibitory activities of these extracts were further verified by applying linear regression analysis to 2 independent experiments, referred to as experiments 1 and 2. The hits were clearly distinguished from nonresponders and active responders, aligning with PKC412-positive controls in the lower left corner of the resulting plots (Figure 2G). Subsequently, the 4 hits were found to exhibit dose-dependent reductions in FGFR3 activities (Figure 2H). Hit 4 is the ethanol extract from *Amaranthus spinosus*, a widely available food plant in Taiwan and tropical countries. Furthermore, the ethanol extract of *Amaranthus mangostanus* has been demonstrated to prevent ovariectomy-induced bone loss in mice (22). Thus, it was selected for further validation and analysis. To assess batch-to-batch consistency of hit 4, we prepared 2 different batches of ethanol extract (hit 4 B1 and hit 4 B2) from plant collected from different locations in Taiwan during different seasons. Both batches displayed inhibitory properties against FGFR3 (Figure 2I). Furthermore, we tested ethanol extracts from 2 plant species closely related to hit 4, *A*. *viridis* (hit 4-1) and *A*. *tricolor* (hit 4-2), which also showed dose-dependent reductions in FGFR3 activation (Figure 2J). These results demonstrate that the R/ATA system could successfully identify hits, and the methods employed for plant extracts preparation were reliable. The consistent inhibitory activity of hit 4 across different batches and related plant species further supports its potential as a reliable and reproducible candidate for further investigation.

The functional effects of hit 4–mediated inhibition of FGFR3 activity were further assessed in a clinically relevant FGFR3-overactivated human MM cell line, KMS-11 cells, which ectopically expressed FGFR3^Y373C^. The viability of KMS-11 cells is dependent on FGFR3 activation (23). Consistent with this, the viability of KMS-11 cells was reduced following treatment with hit 4 and extracts of a closely related species (hit 4-1 and hit 4-2), whereas the extract from a nonresponder did not affect cell viability (Figure 2K). Of note, the reduction in KMS-11 cell viability correlated with decreased FGFR3 accumulation and downregulation of FGFR3 downstream signaling pathway components, including phosphorylated PLCγ (24), PI3K (25), and ERK1/2 (26), observed at 1 hour posttreatment (Figure 2L). These results validate the bioactivity of hit 4, which includes both hit 4-1 and hit 4-2, as it effectively inhibits cell viability and FGFR3 early activation in FGFR3-activated MM cells. *A*. *viridis* (hit 4-1), which is widely cultivated in Taiwan, was used for further bioactive compound discovery.

### Identification of bioactive fractions and compounds from A. viridis extracts.

Standard bioassay-guided sequential fractionation (27) was used to identify the most potent fractions from the ethanol extract of *A*. *viridis* (hit 4-1). During the process of discovering bioactive constituents, the most potent active fractions without cellular toxicity (not affecting the cell/nucleus morphology or cell number) were validated as exerting dose-dependent effects and subjected to further fractionation and purification (Supplemental Figures 2–4). The initial fractionation of *A*. *viridis* is presented in Supplemental Figure 2A. Among the 5 initial fractionations, F3, F4, and F5 exhibited inhibitory activities toward FGFR3 activation, with F4 showing the highest potency (Supplemental Figure 2B). We also demonstrated the batch-to-batch preparation consistency of F4 from 2 different batches of *A*. *viridis* (hit 4-1 B1-F4 and hit 4-1 B2-F4) in inhibiting FGFR3 activity (Supplemental Figure 2C). Overall, the R/ATA system enabled the consistent identification of active fractions from this complex plant extract and validated their bioactivities.

**Figure 3:**
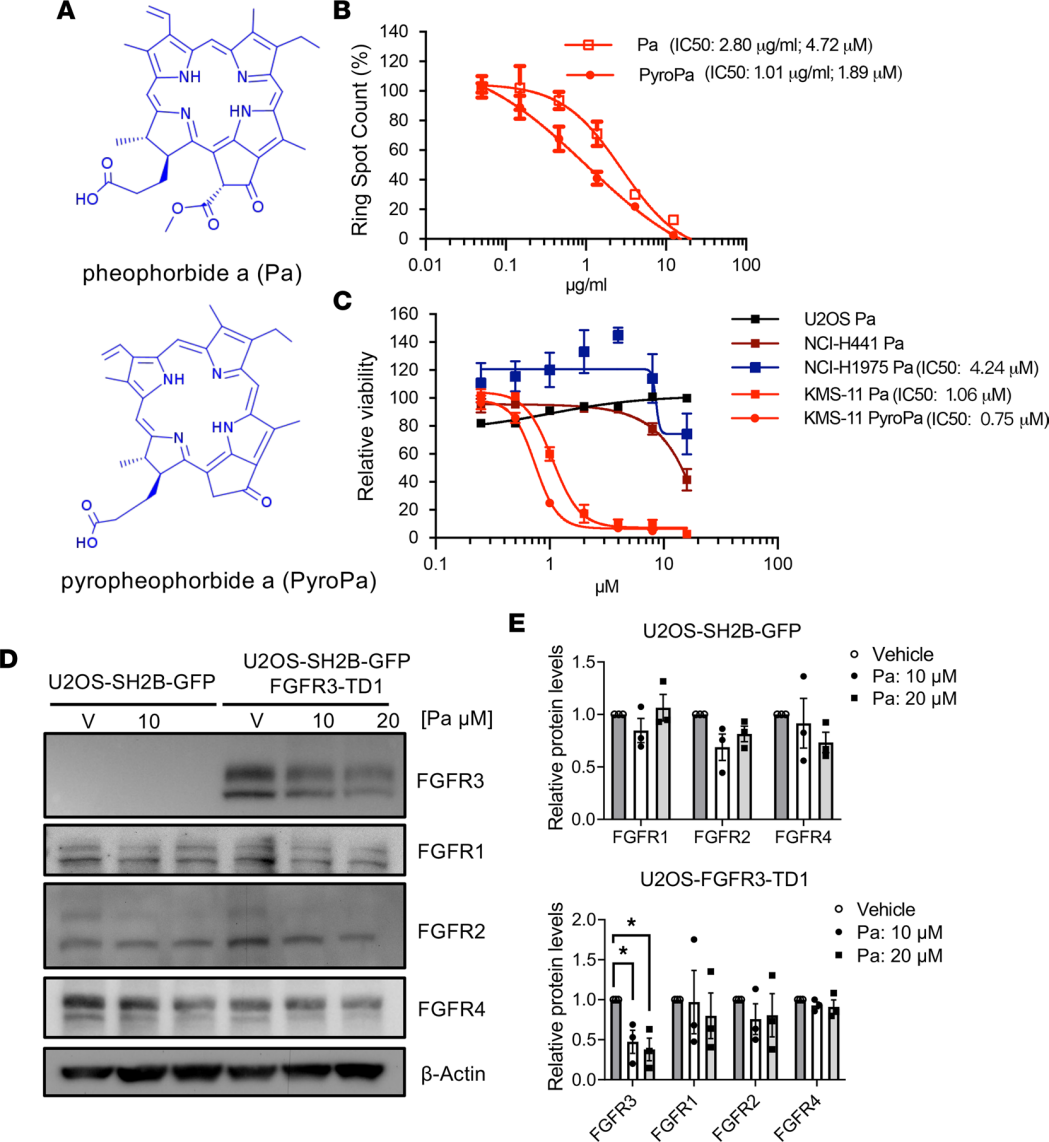
Identification of Pa from the active fraction of *A*. *viridis* as a specific inhibitor of FGFR3 activity. (**A**) The structures of Pa and PyroPa are shown. (**B**) The dose-response curves indicate that Pa and PyroPa inhibit FGFR3 activity using R/ATA system. Data are shown as mean ± SEM of triplicates. (**C**) The effects of Pa on cell viability in various cancer cell lines. Cells were treated with indicated concentrations of Pa or PyroPa for 48 hours. Cell viability was determined by WST-1 assay. Data are presented as mean ± SEM of 3 to 4 independent experiments. (**D**) The effects of Pa on protein levels of the FGFR family members were examined in U2OS-SH2BGFP cells or U2OS-TDI-FGFR3/SH2BGFP cells. Cells were treated with indicated concentration of Pa or vehicle control (V) for 1 hour, and protein levels were analyzed by immunoblotting. (**E**) Relative protein levels for each FGFR family member were quantified and compared with vehicle-treated controls. Data are presented as mean ± SEM of 3 independent experiments. *P < 0.05.

We then sequentially identified the most potent active fraction (Supplemental Figures 2–4) and finally identified 2 active molecules, pheophorbide a (Pa) and pyropheophorbide a (PyroPa) (Figure 3, A and B), present at high purity in the 2 final potent fractions (as demonstrated using HPLC and MS analysis) (Supplemental Figure 4, A–E). In summary, our approach allowed us to identify and characterize 2 active compounds, Pa and PyroPa, from *A*. *viridis*.

We then validated the effects of Pa and PyroPa on inhibition of FGFR3 activity in KMS-11 cells. Both Pa and PyroPa inhibited the viability of KMS-11 cells (Figure 3C). Since Pa and PyroPa are both porphyrins, we further tested a few other porphyrins (Supplemental Figure 5A) and demonstrated that temoporfin and phyrophenophobide a methyl ester (PyroPa methyl ester) could also inhibit FGFR3 activation in the R/ATA system and reduce viability of KMS-11 cells (Supplemental Figure 5, B and C). However, chlorophyllin could only reduce FGFR3 activation and viability of KMS-11 cells at high concentration (Supplemental Figure 5, B and C). Note that the precursor of porphyrins, 5-aminolevulinic acid, did not inhibit FGFR3 activity, and it did not affect viability of KMS-11 cells (Supplemental Figure 5, B and C). We concluded that the shared porphyrin structure in identified active compounds Pa and PyroPa is important for inhibition of FGFR3 activation in the screening system and reduction of cell viability of KMS-11 cells.

To further investigate the specific inhibitory effect of Pa on FGFR3-dependent cancer cell viability, we evaluated Pa impacts in cancer cell lines for which growth is independent of FGFR3 activity. The cell lines included U2OS, NCI-H441, and NCI-H1975 cells; no endogenous FGFR3 expression was detected in any of these lines (RNA HPA cell line gene data, The Human Protein Atlas, https://www.proteinatlas.org/about/download). NCI-H441is a KRAS-dependent lung adenocarcinoma cancer cell line (28), and NCI-H1975 is a non–small cell lung cancer cell line harboring EGFR L858R/T790M activating mutations (29). In contrast with the dose-dependent inhibitory effect of Pa on cell viability in KMS-11 cells, Pa did not have a dose-dependent inhibitory effect on cell viability in U2OS, NCI-H441, or NCI-H1975 cells (Figure 3C). Only at high doses of Pa did we observe a partial reduction in cell viability in NCI-H441 and NCI-H1975 cells (Figure 3C). These results suggest that Pa exhibits greater potency in inhibiting viability of cancer cells dependent on ectopic expression of FGFR3 compared with cells harboring mutations in RAS family or other receptor tyrosine kinases, such as EGFR.

We next examined the selectivity of Pa for members of the FGFR family. U2OS-SH2B-GFP cells endogenously express FGFR1, 2, and 4. Therefore, we treated U2OS-SH2B-GFP cells without or with stable expression of an FGFR3 activation mutant (U2OS-FGFR3-TDI) with vehicle or Pa and determined protein levels of FGFRs by Western blot analysis. In Pa-treated cells, the FGFR3 protein level was dramatically reduced; however, Pa had no significant inhibitory effect on other FGFR family members, as shown in the quantitative results (Figure 3, D and E). Thus, we concluded that Pa has the highest potency to reduce FGFR3 accumulation.

Up to this point in the study, we had identified active compounds, Pa and PyroPa, from *A*. *viridis* and showed that the shared porphyrin structure is important for inhibition of FGFR3 activity. Furthermore, we found that Pa has the highest potency toward FGFR3 among FGFR family members.

### Pa-mediated inhibition of FGFR3 signaling and its downstream effects in FGFR3-overactivated MM cells.

**Figure 4:**
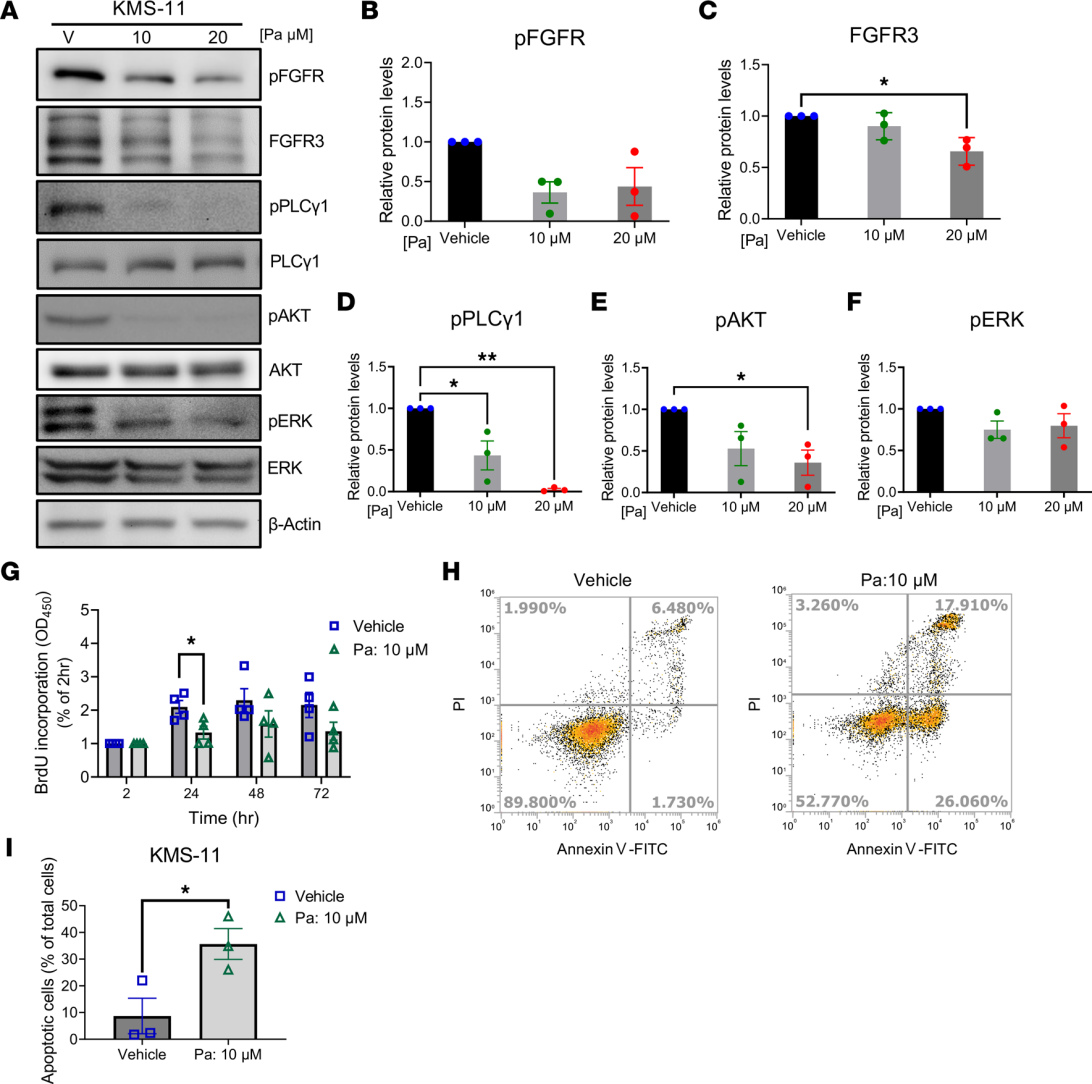
Pa reduces FGFR3 signaling, decreases cell proliferation, and induces cell apoptosis processes in MM cells. (**A**) Pa treatment reduced FGFR3 accumulation and its downstream signaling in KMS-11 cells. KMS-11 cells were treated with vehicle or indicated concentration of Pa for 1 hour. The levels of total and phosphorylated FGFR3 and its downstream effectors were analyzed by immunoblotting. (**B**–**F**) The relative levels of phosphorylated FGFR3 and downstream effectors were normalized to the corresponding total protein levels and compared with vehicle-treated controls. Data are shown as mean ± SEM of 3 independent experiments. (**G**) Pa treatment reduced KMS-11 cell proliferation. KMS-11 cells were treated with vehicle or Pa for the indicated times, and cell proliferation was analyzed by 5′-bromo-2′-deoxyuridine (BrdU) incorporation assay. Data are shown as mean ± SEM of 4 independent experiments. (**H** and **I**) KMS-11 cells were treated with vehicle or Pa for 8 hours and stained by annexin V and PI; the proportion of apoptotic cells was analyzed by flow cytometry. (**H**) Represented data are shown. (**I**) The percentage of apoptotic cells from 3 independent experiments. Data are shown as mean ± SEM. (**B**–**F**) Statistical significance was determined by 1-way ANOVA with Tukey’s multiple-comparison test or (**G** and **I**) 2-tailed Student’s *t* test. *P < 0.05, **P < 0.01.

In our next experiments, we evaluated Pa-induced inhibition of FGFR3 downstream signaling, which regulates diverse biological activities. It is known that ectopic FGFR3 expression in MM cells, such as KMS-11, induces cell proliferation and blocks apoptosis through ERK signaling and the PI3K/AKT signaling pathway (25, 30–33). Accordingly, we showed that treatment of KMS-11 cells with Pa dramatically reduced FGFR3 protein accumulation and inhibited FGFR3 activation–induced phosphorylation of downstream effectors, including PLCγ1, AKT, and ERK (Figure 4, A–F). Most importantly, Pa inhibited proliferation and antiapoptosis processes that are promoted by FGFR3 activation, as demonstrated by a BrdU incorporation assay and FITC-annexin V/propidium iodide (PI) apoptosis assay (Figure 4, G–I). Taken together, these findings revealed that Pa can effectively reduce FGFR3 accumulation and FGFR3 downstream signaling pathways, which leads to suppression of cell proliferation and induction of apoptosis in MM cells.

### Pa-induced reduction of FGFR3 half-life in FGFR3-activated MM cells and chondrocytes.

**Figure 5:**
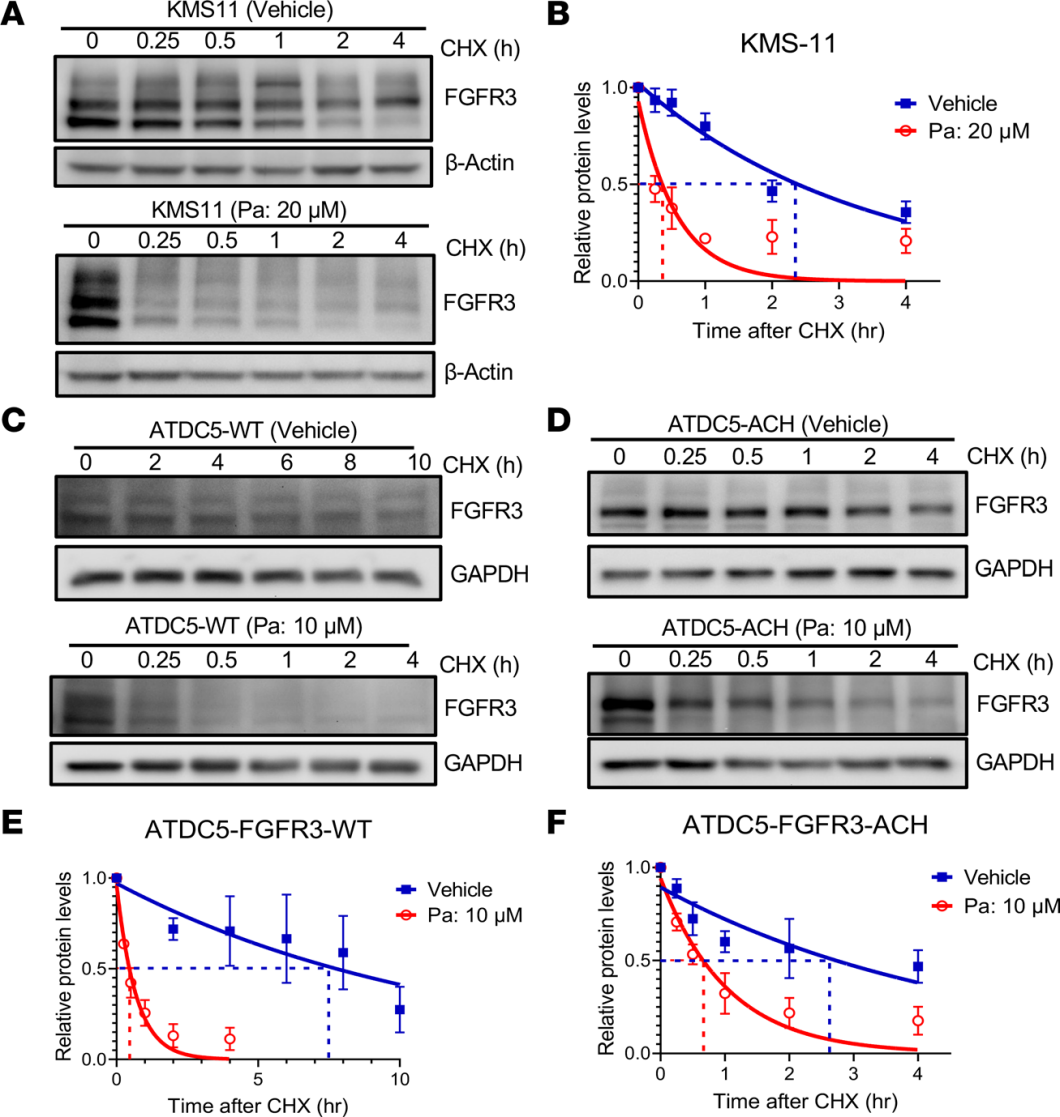
Pa compromises FGFR3 protein stability in FGFR3-activated MM cells and chondrocytes. (**A** and **B**) KMS-11 cells were treated with either vehicle or 20 μM Pa in the presence of 80 μg/mL cycloheximide (CHX) for the indicated times. (**A**) Protein levels were detected by immunoblotting. (**B**) The relative levels of FGFR3 protein were quantified, normalized to β-actin, and compared with time 0 of CHX treatment. (**C**–**F**) WT and ACH-FGFR3–expressing ATDC5 cells were treated with vehicle or 10 μM Pa in the presence of 100 μg/mL CHX for indicated times. (**C** and **D**) Protein levels were detected by immunoblotting. (**E** and **F**) The relative protein levels were quantified, normalized to GAPDH, and compared with time 0 of CHX treatment. (**B**, **E**, and **F**) Data are shown as mean ± SEM of 3 to 4 independent experiments.

We then elucidated the mechanism by which Pa inhibits FGFR3 activity. We observed a reduction in FGFR3 protein levels when KMS-11 cells were treated with Pa for 1 hour (Figure 4, A and C). Activating mutations of FGFR3 have been shown to increase its stability and prolong its half-life (34). Thus, we hypothesized that Pa might reduce FGFR3 accumulation by regulating protein stability. To test this possibility, we determined the half-life of FGFR3 in KMS-11 cells treated with vehicle or Pa in the presence of protein synthesis inhibitor cycloheximide (CHX). The half-life of FGFR3 in Pa-treated KMS-11 cells was shorter than that in vehicle-treated controls (Figure 5, A and B), suggesting that Pa compromises FGFR3 stability in FGFR3-activated MM cells.

To begin addressing the possibility that Pa might be able to functionally alter ACH signs and symptoms, we first tested whether Pa can also compromise FGFR3 stability in chondrocytes. For this purpose, we established ATDC5 cells, a chondrocyte cell line, that stably expresses WT FGFR3 or the G380R mutant (ACH) of FGFR3. Similar to our results in MM cells, Pa reduced the protein stability of both WT and ACH FGFR3 in ATDC5 cells (Figure 5, C–F). To further investigate the impact of Pa on the stability of FGFR3 in a clinically relevant system, we conducted experiments in chondrocytes isolated from *FGFR3^ACH^* mice. Pa significantly reduced the half-life of FGFR3 in chondrocytes from *FGFR3*^ACH^ mice, as compared with vehicle-treated controls (Supplemental Figure 6). These results demonstrated that Pa reduces FGFR3 stability in chondrocytes. Thus, our data reveal that Pa can reduce FGFR3 stability in both MM cells and chondrocytes.

To explore the molecular mechanisms by which Pa reduces FGFR3 stability, we examined a known mechanism regulating FGFR3 stability. Activating mutations have been shown to disrupt FGFR3 protein ubiquitination, which acts as a signal for targeted protein degradation (34, 35). We therefore investigated whether Pa could increase FGFR3 ubiquitination to induce protein degradation. Unexpectedly, less ubiquitination on FGFR3 was observed in KMS-11 cells treated with Pa (Supplemental Figure 7A). We then tested whether Pa could affect ubiquitin-independent macroautophagy protein degradation by examining the conversion of an autophagosomal marker, the cytosolic form of microtubule-associated protein 1A/1B-light chain 3 (LC3-I), to its lipidated form, LC3-II (36). No difference in LC3 conversion was observed in KMS-11 cells treated with or without Pa, according to Western blotting (Supplemental Figure 7B). Thus, it is unlikely that Pa reduces FGFR3 stability by increasing FGFR3 ubiquitination or acting on the macroautophagy protein degradation pathway.

We demonstrated that Pa reduced FGFR3 protein abundance by shortening its half-life, which subsequently attenuated FGFR3 downstream signaling in both cancer cells and chondrocytes. In some cases, drugs that perturb specific signaling targets can induce positive or negative feedback loops that may alter the expression of the target and compromise long-term drug efficacy (37). As such, we probed the effects of Pa on FGFR3 gene expression. We found that after treatment with Pa, the mRNA levels of FGFR3 remained relatively low in both FGFR3-activated MM cells and chondrocytes (Supplemental Figure 8, A–C). These results suggest that Pa is unlikely to induce positive feedback leading to an increase in gene transcription.

### Pa-induced rescue of defective growth in cultured femurs from FGFR3^ACH^ mice.

**Figure 6:**
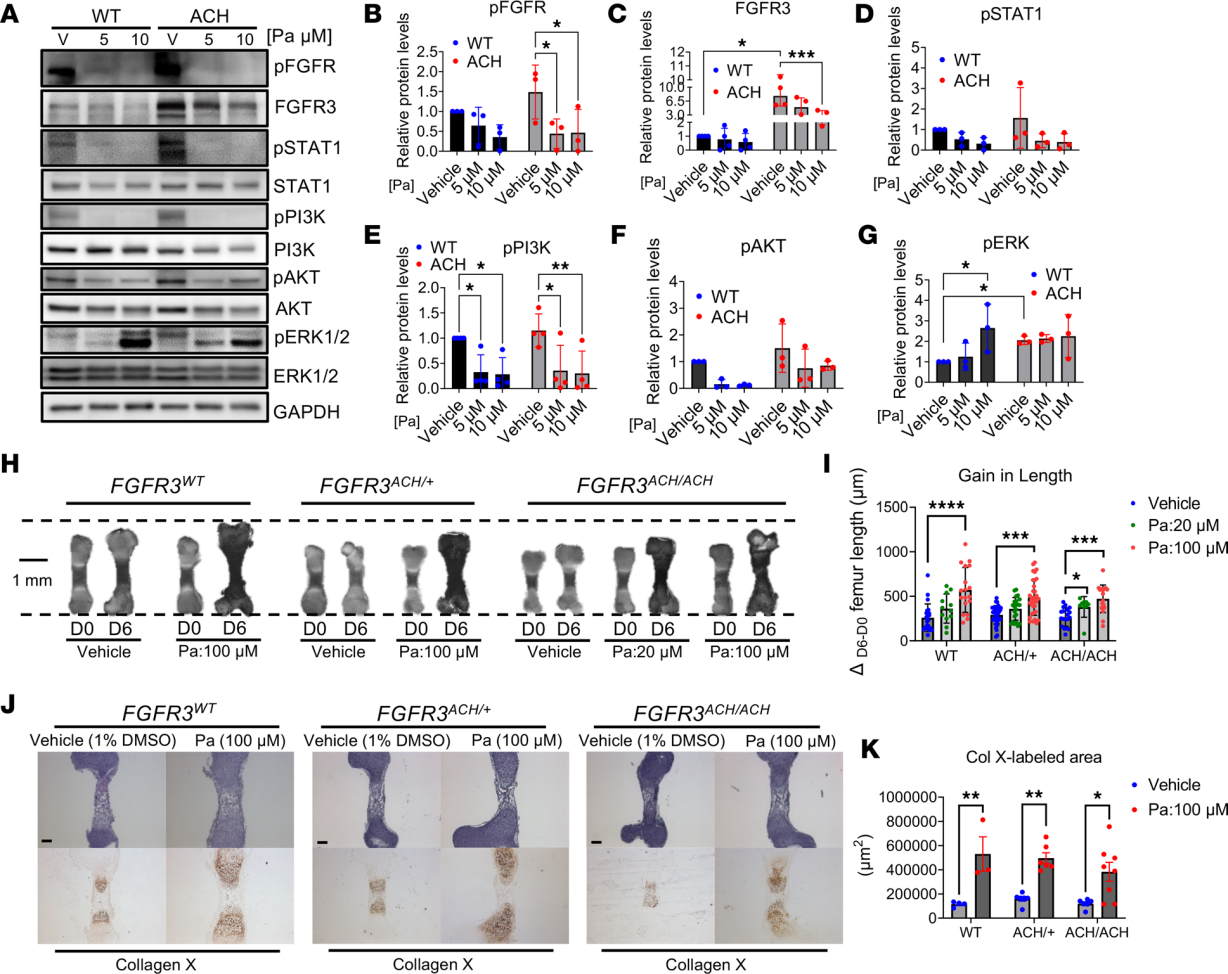
Pa counteracts overactive FGFR3 signaling in chondrocytes and rescues defective growth of cultured femurs from *FGFR3^ACH^* mice. (**A**) ATDC5 cells stably expressing either WT or ACH FGFR3 were treated with vehicle or the indicated concentrations of Pa in the presence of FGF2 (20 ng/mL) for 2 hours. Levels of total and phosphorylated FGFR3 and downstream effectors were examined by immunoblotting. (**B**–**G**) The relative phosphorylated protein levels were normalized to the corresponding total protein levels and compared with vehicle-treated controls. Data are shown as mean ± SEM of 3 to 4 independent experiments and were analyzed by 1-way ANOVA with Tukey’s multiple comparison; *P < 0.05, **P < 0.01, ***P < 0.001. (**H**–**K**) Femurs from WT, *FGFR3^ACH/+^*, and *FGFR3^ACH/ACH^* mice at E16.5 were isolated and cultured for 6 days in the presence of vehicle or indicated concentrations of Pa. (**H**) Representative images of E16.5 femurs before and after 6 days of culture are shown. Scale bar: 1 mm. (**I**) The increased femur lengths after treatment were calculated. Data are expressed as mean ± SD (*n* = 9–35) and analyzed by 1-way ANOVA. *P < 0.05, ***P < 0.001; ****P < 0.0001. (**J**) Representative images of H&E-stained and collagen X–stained femurs cultured for 6 days. Scale bar: 200 μm. (**K**) Quantification of the collagen X–stained area in the femurs. Data are shown as mean ± SEM; *n* = 3–8. Statistical significance was determined by Student’s 2-tailed *t* test. *P < 0.05 and **P < 0.01.

To further assess the therapeutic potential of Pa for ACH, we determined how Pa treatment affects FGFR3 downstream signaling in chondrocytes using ATDC5 cells expressing WT or ACH FGFR3. In ACH pathogenesis, FGFR3 activation suppresses chondrocyte proliferation and differentiation in the growth plate, thereby limiting long bone growth (38). It is known that FGFR3 activation inhibits chondrocyte proliferation via activation of STAT1 (39), and it inhibits chondrocyte differentiation through activation of MAPK/ERK signaling (40, 41). Meanwhile, FGFR3 activation negatively modulates chondrocyte apoptosis through activation of PI3K/AKT signaling (42). Correspondingly, ACH FGFR3 expression in ATDC5 cells increased FGFR3 accumulation and activation, which induced the phosphorylation of STAT1, ERK1/2, PI3K, and AKT (Figure 6, A–G). Treatment of the cells with Pa suppressed FGFR3 accumulation and phosphorylation, along with FGFR3 activation–induced phosphorylation of STAT1, PI3K, and AKT in both WT and ACH FGFR3–expressing ATDC5 cells (Figure 6, A–F). However, Pa induced ERK1/2 phosphorylation in WT FGFR3–expressing cells (Figure 6, A and G). Overall, these results suggest that Pa can partially reverse FGFR3 activation–induced signaling events that are known to impact chondrocyte proliferation, differentiation, and apoptosis.

We therefore directly evaluated the therapeutic potential of Pa on defective long bone growth using ex vivo femur cultures of WT and *FGFR3^ACH^* mice (17). Mouse femurs were isolated at embryonic day 16.5 (E16.5) and cultured in the presence or absence of Pa for 6 days. Pa markedly increased the femur length gains in WT, *FGFR3^ACH/+^*, and *FGFR3^ACH/ACH^* mice. Importantly, the Pa-increased femur length gains in *FGFR3^ACH/ACH^* tissues occurred in a dose-dependent manner (Figure 6, H and I). Most significantly, we noticed that Pa increased the area of hypertrophic chondrocytes in the femurs, according to histological analysis of H&E-stained tissues. Hypertrophic chondrocytes are considered to be in a terminal state of chondrocyte differentiation and are essential for long bone growth; the cells exhibit characteristic high levels of type X collagen expression (14, 40). We therefore immunostained hypertrophic chondrocytes in the femurs for type X collagen. Pa treatment significantly increased the area of the hypertrophic zone in femurs across all genotypes, as compared with vehicle-treated controls (Figure 6, J and K). These results suggest that Pa enhances chondrocyte differentiation and that Pa can reverse FGFR3 activation signaling in chondrocytes to promote long bone growth in femur cultures.

## Discussion

Gain-of-function mutations in FGFR3 contribute to the pathogenesis of cancers and skeletal dysplasias, making it a vital therapeutic target. Nevertheless, there remains a lack of specific targeting approaches for FGFR3 (10, 13). Since drug discovery using plant-derived libraries benefits from the vast structural diversity of natural products, it is considered an appealing strategy (16). In this work, we developed a receptor/adaptor translocation assay system for screening FGFR3-inactivating molecules (called R/ATA), and we used it to screen complex mixtures of natural products. By doing so, we were able to identify and characterize FGFR3-inhibiting molecules from the most potent bioactive fractions of *A*. *viridis* extract. One identified compound, Pa, was shown to reduce FGFR3 half-life and attenuate FGFR3 signaling in FGFR3-activated MM cells and chondrocytes. Most importantly, Pa displayed therapeutic potential, as it could promote femoral growth in cultured bones of *FGFR3^ACH^* mice. Together, these results introduce an effective platform for identification of FGFR3 modulators that can be developed into potential treatments for FGFR3-related diseases.

The discovery of bioactive compounds from natural products is often hampered by methodological difficulties in precisely measuring the downstream signaling of drug targets and by the limited availability of cell-based assays for screening complex mixtures of natural compounds (16). Here, we established the R/ATA system, a high-throughput cell-based receptor/adaptor translocation imaging system, to identify compounds that inhibit early events of FGFR3 activation from complex mixtures of natural products. Our system utilizes U2OS cells, which endogenously express 3 FGFRs but not FGFR3. In these cells, we expressed an adaptor protein SH2(SH2B) that specifically interacts and internalizes with a coexpressed form of activated FGFR3. We established the R/ATA system using U2OS cells expressing SH2(SH2B)/GFP and TDI-FGFR3, which form stable dimers with constitutive kinase activation that recruit SH2(SH2B)/GFP. These molecular actions result in an internalized protein complex that can be tracked as an intracellular spot pattern. In the R/ATA system, disruption of early events of FGFR3 activation can be quantified according to reductions in the number of internalized spots at 1 hour after treatment. Thus, it is capable of identifying compounds that modulate early events of FGFR3 activation. Importantly, the R/ATA can specifically report FGFR3 activation, and the readout is not prone to interference by autofluorescence or fluorescence-quenching compounds. Therefore, this system is well suited for screening modulators from complex mixtures of natural products, such as herbal extracts. This system is capable of reporting potential cellular toxicity of the extracts, according to changes in cell and nucleus morphology as well as a reduced number of cells imaged during screening. These parameters provide valuable insights into the potential toxic effects of the tested extracts on cellular health and viability. A recent report described another system for quantification of FGFR3 signaling that tracks recruitment of a downstream signal transducer, GRB2, to FGFR3 at the plasma membrane of living cells (43). However, that system is not designed for drug screening purposes. Thus, our R/ATA provides a platform for identification of new treatments for FGFR3-mediated diseases.

We used the R/ATA system to identify Pa as a factor that can reduce FGFR3 protein abundance by directly shortening its half-life, which attenuates FGFR3 downstream signaling in both cancer cells and chondrocytes. Importantly, Pa did not induce FGFR3 mRNA expression, suggesting that Pa likely causes long-term reductions of FGFR3 levels through its effects on protein stability, without activating positive feedback signaling for increased gene transcription. Consistently, Pa effectively suppresses FGFR3 signaling–induced clinical phenotypes in cancer cells and femurs. These results suggest that Pa has great potential for further development into a treatment for diseases that result from FGFR3 overactivation. Potential disease targets include FGFR3-related skeletal dysplasias and also other conditions caused by excessive FGFR3 function, such as cancers and seborrheic keratoses.

Intriguingly, Pa is a product of chlorophyll breakdown. Chlorophyll and its derivatives have a long history of use in traditional medicine, as they exhibit a broad range of biological activities, including antioxidant and immunostimulatory properties (44). However, the mechanisms underlying the beneficial effects of chlorophyll and its derivatives are not well defined. Pa has also been used as a photosensitizer and in combination with light to generate reactive oxygen species that can directly kill tumor cells (45, 46). Our study reveals a role of Pa in reducing FGFR3 signaling. Although the mechanism by which Pa reduces FGFR3 half-life remains to be elucidated, further study on this topic may enhance our understanding of the fundamental processes underlying FGFR3 regulation and protein stability.

In this study, we provide a proof of concept that Pa improves long bone growth using ex vivo femur cultures. However, testing the efficacy of Pa in ACH mice is currently not feasible due to its low aqueous solubility. Further optimization of the compound is necessary to improve its solubility and potency. Additionally, the specific targeting of drugs to chondrocytes in developing bone holds great promise for enhancing therapeutic effects while minimizing potential toxicity and side effects. One potential targeting approach is the use of multiarm avidin nano-constructs, which have been designed for intracartilage administration of small-molecule drugs to specifically target articular chondrocytes for the treatment of osteoarthritis (47).

In summary, we have established and validated a cell-based protein translocation system for monitoring FGFR3 activation, which greatly facilitates the identification and purification of FGFR3 inhibitory molecules from complex mixtures. Using this system, we identified bioactive compounds from a plant extract that can inhibit FGFR3 activation. Finally, we validated the applicability of this approach to drug discovery by demonstrating the therapeutic potential for the identified molecules in models of FGFR3-driven disease.

## Methods

### Cell culture.

The U2OS human osteosarcoma cell line was provided by Klim King (Institute of Biomedical Sciences, Academia Sinica, Taiwan) and was maintained in McCoy’s 5A medium (complete medium; Invitrogen) supplemented with 10% fetal bovine serum (FBS) in a humidified incubator at 37°C and 5% CO_2_. KMS-11 cells (Japanese Collection of Research Bioresources, JCRB1179) were maintained in RPMI 1640 medium (Themo Fisher Scientific) supplemented with 10% FBS. NCI-H441 and NCI-H1975 cell lines were provided by Han-Chung Wu (Institute of Cellular and Organismic Biology, Academia Sinica, Taiwan) and cultured in RPMI 1640 medium supplemented with 10% FBS. The ATDC5 cell line was provided by Chien-Chang Chen (Institute of Biomedical Sciences, Academia Sinica, Taiwan). ATDC5 cells were maintained in DMEM/F12 medium (Themo Fisher Scientific) containing 5% FBS.

### Plasmid construction and site-directed mutagenesis.

The full-length cDNA for Renilla reniformis GFP was subcloned into the pcDNA3.1/Hygro expression vector (Invitrogen). SH2 proteins fused to the N-terminus of GFP were generated by subcloning the SH2 domain of human SH2Bβ and the SH2 domain of human PLCγ into the GFP expression vector. Full-length cDNA for human FGFR3 (OriGene) was subcloned into the pcDNA3.1 expression vector (Invitrogen). Mutations were introduced into FGFR3 by oligonucleotide-directed mutagenesis with the QuikChange Site-Directed Mutagenesis kit (Stratagene). Plasmids were transfected into cells using Lipofectamine 3000 (Invitrogen) according to the manufacturer’s protocol.

### Immunoprecipitation and immunoblotting.

For immunoprecipitation experiments, cell lysates were incubated with an anti-FGFR3 antibody along with protein A/G PLUS-Agarose (sc-2003, Santa Cruz Biotechnology). The immunoprecipitated samples were analyzed by immunoblotting using RrGFP (Santa Cruz Biotechnology, sc-9996) and FGFR3 (Cell Signaling Technology, 4574) antibodies. For experiments not requiring immunoprecipitation, lysates were used for immunoblotting with primary antibodies against FGFR1 (ABclonal, A13493), FGFR2 (Santa Cruz Biotechnology, sc-6930), FGFR3 and FGFR4 (Santa Cruz Biotechnology, sc-136988), p-FGFR (Tyr653/654) (Cell Signaling Technology, 3476), p-PLCγ1 (Tyr783) (Cell Signaling Technology, 2821), p-PI3K p85α (Tyr508) (Santa Cruz Biotechnology, sc-12929), p-AKT (Ser473) (Cell Signaling Technology, 4060), p-STAT1 (Tyr701) (Cell Signaling Technology, 9167), p–p44/42 MAPK (Erk1/2) (Thr202/Tyr204) (Cell Signaling Technology, 4376), PLCγ1 (Cell Signaling Technology, 2822), PI3K p85 (Cell Signaling Technology, 4257), STAT1 (Cell Signaling Technology, 14994), AKT (pan) (Cell Signaling Technology, 4691), p44/42 MAPK (Erk1/2) (Cell Signaling Technology, 9102), β-actin (GeneTex, GTX109639), and GAPDH (Proteintech, 10494-1-AP). The band intensities were quantified by VisionWorksLS Analysis Software.

### Preparation of U2OS cells for high-content imaging screening.

For high-content screening, all steps of sample plate preparation, including compound treatment, fixation, and plate washing, were fully automated and performed using an EL405uv system (Bio-Tek Instruments). U2OS cells stably expressing FGFR3 and SH2-GFP were seeded at a density of 4 × 10^3^ cells/well in black, 96-well Packard Viewplates; incubated overnight in complete medium at 37°C in a CO_2_ incubator; and then transferred to 100 μL serum-free medium containing plant extracts or fractions with a final concentration of 1% DMSO. After 1 hour at 37°C in a CO_2_ incubator, cells were fixed with 4.5% formaldehyde, and nuclei were labeled with Hoechst staining (MilliporeSigma). Cells treated with vehicle (DMSO) or 4 μM PKC412 (Cayman Chemical) were used for protocol optimization.

### Imaging and analysis on an ArrayScan VTI HCS system.

Cell images were automatically obtained using an ArrayScan VTI HCS Reader (Cellomics). Filter sets appropriate for detection of the 2 fluorophores were used. A 20× objective with 0.4 numerical aperture was used for imaging. The accompanying Compartmental Analysis BioApplication (Cellomics) software was used to analyze the images after optimizing protocol settings within the application. Hoechst-labeled nuclei were used to identify individual cells, and for each cell, the nucleus (circle) and cytoplasmic area (ring) were defined by the parameters set in the software. The GFP spots in the ring region were detected, and the spot number count (ring spot count) and spot intensity (ring spot intensity) were measured.

### Plant extract preparation, partitioning, and bioassay-guided fractionation.

To generate ethanol extracts, plants collected in Taiwan were dried and ground into powder. The ground powders were extracted using 95% ethanol. The ethanol extracts were dried and dissolved in DMSO to a final concentration of 37 mg/mL. For preliminary screening, ethanol extracts from whole plants of 101 different species from 75 families were tested at a uniform concentration of 10 μg/mL in 1% DMSO. The ethanol extracts of a second batch of hit 4 (*A*. *spinosus*) and 2 closely related species, *A*. *viridis* (hit 4-1) and *A*. *tricolor* (hit 4-2), were prepared using the same method. Extracts were condensed and stored at 4°C before further fractionation. Large-scale preparations of ethanol extracts were made from *A*. *viridis* by the Industrial Technology Research Institute, Hsinchu, Taiwan, for further purification. The ethanol extract was fractionated as illustrated in Supplemental Figure 2. The procedures used for further fractionation and purification are described in the Supplemental Methods.

### Treatment of KMS-11 cells.

KMS-11 cells were treated with the indicated drugs, plant extracts, or vehicle control at the indicated concentrations and times in the presence of heparin (MilliporeSigma, H3149) and FGF2 (R&D Systems, 233-FB) as indicated in the figure legends.

### Cell viability assay.

Cell viability was determined by a colorimetric assay based on the cleavage of the tetrazolium salt WST-1 by mitochondrial dehydrogenases (Roche).

### Identification of bioactive compounds from A. viridis that inhibit FGFR3 activation.

The most potent FGFR3-inhibiting active fractions were further fractionated. The main chemical constituents of 2 final active fractions with high purity (as determined using HPLC and MS analysis) were identified by AnalytiCon Discovery, BRAIN Group, Potsdam, Germany. NMR was used to identify the main chemical constituent of the C2156-N3 as Pa and the main constituent of the C2156-N5 fraction as PyroPa. Pa was then purchased from Cayman Chemical (catalog 16072), and PyroPa was purchased from Santa Cruz Biotechnology Animal Health (sc-264178).

### CHX chase assay.

Cells were incubated with CHX (MilliporeSigma, C1988) in the presence of vehicle or Pa for indicated times. Protein extracts were prepared using 1× RIPA buffer containing protease and phosphatase inhibitors, then subjected to immunoblot analysis. Each experimental condition was performed 3 to 4 times. The results are shown as the average of 3 to 4 independent experiments.

### Cell proliferation assay.

Cell proliferation was determined using a BrdU cell proliferation assay kit (Cell Signaling Technology, 6813) according to the manufacturer’s protocol. Cells were seeded at a density of 5 × 10^4^ cells/well in 96-well plates, then treated with vehicle or the indicated drug. Proliferation of cells was analyzed at the indicated times. Each experimental condition was performed 4 times. The results are shown as the average of 4 independent experiments.

### Flow cytometry analysis for detection of apoptotic cells.

Early apoptosis was detected using FITC-Annexin V Apoptosis Detection Kit I (BD Biosciences), according to the manufacturer’s protocol. Briefly, cells were treated with vehicle or Pa for 8 hours and then harvested, washed with cold PBS, and resuspended in 1× Annexin V Binding buffer. Cells were then subjected to FITC-Annexin V and PI staining for 15 minutes at room temperature. The samples were analyzed using flow cytometry (Thermo Fisher Scientific Attune NxT) using the BL1 (FITC) channel and YL1 (PI) channel. Each experimental condition was performed in triplicate. The results are shown as the average of 3 independent experiments.

### Mouse management.

Mice were housed in a temperature- and humidity-controlled room with a 12-hour light/12-hour dark cycle under specific pathogen–free conditions. All animal protocols were approved by the institutional animal care and use committee at Academia Sinica, Taiwan (Protocol 17-09-1108). The generation of ACH (*FGFR3^ACH^*) mice on a 129Sv background was previously described (17).

### Assessment of long bone growth.

Femurs were isolated from E16.5 embryos and cultured in the DMEM containing 0.2% BSA, 0.5 mM l-glutamine, 40 U/mL penicillin/streptomycin, 0.05 mg/mL ascorbic acid, and 1 mM β-glycerophosphate in the presence of 100 ng/mL FGF2 and treated with vehicle (1% DMSO) or Pa at indicated concentrations for 6 days. The culture medium was changed daily. Imaging was conducted on day 0 and day 6 using a Leica M205 stereomicroscope. For histology and immunohistochemistry (IHC), femurs were fixed in 4% formaldehyde and then processed by the Taiwan Mouse Clinic for decalcification and paraffin embedding. Sections were prepared at 5 μm and examined with H&E staining or IHC staining using a primary antibody against collagen X (Abcam). IHC signal was generated using a DAB Peroxidase Substrate Kit (Vector Laboratories). Femur lengths and areas of hypertrophic chondrocytes were quantified with ImageJ (NIH).

### Statistics.

Statistical analyses were performed using GraphPad Prism 9. Data are presented as mean ± SEM. For analysis of 2 groups, paired 2-tailed Student’s *t* tests were carried out. For multiple groups treated with different dosages, 1-way ANOVA followed by Tukey’s test for multiple comparisons was used. *P* values less than 0.05 were considered statistically significant.

### Study approval.

All animal protocols were in accordance with the institutional authorities’ guidelines and formally approved by Academia Sinica Institutional Animal Care and Utilization Committee, Academia Sinica, Taiwan (Protocol ID: 17-09-1108).

### Data availability.

Values for all data points in graphs are reported in the Supporting Data Values file.

## Author contributions

YCL conceived and supervised the work. YCL and YWL designed the experiments, analyzed the data, interpreted the data, and wrote the manuscript. YCL, YWL, HJK, and WTC performed the experiments and analyzed the data. CFK, JYW, and YTC provided scientific guidance and insights. All authors reviewed, revised, and approved the manuscript.

## Supplementary Material

Supplemental data

Supporting data values

## Version 1 10/12/2023

In-Press Preview

## Version 2 11/22/2023

Electronic publication
