# Supplementary material for: Cell-based screen identifies porphyrins as FGFR3 activity inhibitors with therapeutic potential for achondroplasia and cancer
Source: JCI Insight. 2023 Nov 22;8(22):e171257. doi: 10.1172/jci.insight.171257 (PMC10721322; doi:10.1172/jci.insight.171257)
Supplement: Supplemental data [file jciinsight-8-171257-s114.pdf]

Supplementary Data

Supplemental Materials and Methods

Cell culture and transfections

The U2OS human osteosarcoma cell line was maintained in McCoy's 5A medium (complete medium; Invitrogen, Carlsbad, CA, USA) supplemented with 10% fetal bovine serum (FBS) in a humidified incubator at 37°C and 5% CO_2. For transient transfection assays, plasmids encoding GFP, or SH2(SH2B)/GFP or SH2(PLCγ)/GFP fusion proteins were transfected into U2OS cells one day before experimentation. U2OS cells stably expressing SH2(SH2B)/GFP were transfected with plasmids and selected in the presence of 400 μg/ml Hygromycin B (Invitrogen) for 2 weeks. Thereafter, individual positive clones were isolated by FACS (fluorescence-activated cell sorting) and grown under continued selection to yield individual transfected cell lines. A U2OS cell line stably expressing SH2(SH2B)/GFP was used for subsequent transfection with FGFR3. Plasmids encoding various FGFR3 isoforms (WT, TDI, ACH) or empty expression vector were transfected into the U2OS-SH2(SH2B)/GFP stable cell line and selected with 800 μg/ml G418 (Sigma-Aldrich, St. Louis, MO, USA) for 2 weeks. All transfections in U2OS cells were carried out with Effectene (Qiagen, Hilden, Germany), according to the manufacturer's instructions.

Plasmid construction and site-directed mutagenesis

The full-length cDNA for *Renilla reniformis* green fluorescent protein (*GFP*) was amplified by polymerase chain reaction (PCR) and subcloned into the pcDNA3.1/Hygro expression vector (Invitrogen) using NotI and XbaI sites. SH2 proteins fused to the N-terminus of GFP were generated by amplifying the Src homology 2 (SH2) domain of human SH2Bβ and the SH2 domains of human PLCγ from genomic DNA and subcloning them into BamHI and NotI sites in the GFP expression vector. Full-length cDNA for human *FGFR3* (OriGene, Rockville, MD, USA) was subcloned into the BamHI and XbaI sites of the pcDNA3.1 expression vector (Invitrogen). Mutations were introduced into *FGFR3* by oligonucleotide-directed mutagenesis using the QuikChange Site-Directed Mutagenesis kit (Stratagene, La Jolla, CA, USA). The mutagenic oligonucleotides used are as follows: Y373C (TDI), 5′-cga ggc ggg cag tgt gtg tgc agg cat-3′ (forward) and 5′-atg cct gca cac aca ctg ccc gcc tcg-3′ (reverse); G380R (ACH), 5′-gca tcc tca gct aca ggg tgg gct tct tc-3′ (forward) and 5′-gaa gaa gcc cac cct gta gct gag gat gc-3′ (reverse). All plasmids were verified by DNA sequencing.

Immunoprecipitation and immunoblotting

Cells were lysed in lysis buffer (50 mM Tris-HCl pH 8.0, 150 mM NaCl, 5 mM EDTA, 0.5% NP-40) supplemented with protease inhibitor cocktail (Roche, Mannheim, Germany). For immunoprecipitation experiments, cell lysates were pre-cleared with protein A/G PLUS-Agarose (sc-2003; Santa Cruz Biotechnology, Santa Cruz, CA, USA) and then incubated with 2 μg of a polyclonal anti-FGFR3 antibody (C-15; Santa Cruz Biotechnology) for 1 h at 4°C. Protein A/B beads were then added and incubated overnight at 4°C. The immunoprecipitated samples were washed four times with lysis buffer and analyzed by immunoblotting. For experiments not requiring immunoprecipitation, lysates were immunoblotted with antibodies. The signals were detected using enhanced chemiluminescence (ECL; Amersham Biosciences, Little Chalfont, Buckinghamshire, UK).

U2OS cell preparation for high-content imaging screening

For high-content screening, all steps of sample-plate preparation, including compound treatment, fixation, and plate washing, were fully automated and were performed using a Bio-Tek EL405uv system (Bio-Tek Instruments, Inc., Winooski, VT, USA). U2OS FGFR3-TD1/SH2-GFP were seeded at a density of 4×10^3 cells/well in black 96-well Packard Viewplates, incubated overnight in complete medium at 37°C in a CO_2 incubator, and then transferred to 100 μl serum-free medium containing PKC412 or plant extract. The final DMSO concentration was 1%. After incubating for 1 h at 37°C in a CO_2 incubator, cells were fixed with 4.5% formaldehyde and cell nuclei were labeled with a 2 μg/ml Hoechst stain solution (Sigma-Aldrich). Cells treated with either control (DMSO) or 4 μM PKC412 were used for protocol optimization.

Plant extract preparation

To generate ethanol plant extracts, the aerial parts of plants were collected and washed with water. Then whole plants were dried in a 65°C oven for 13 h and ground into powder. The ground powders were extracted in a 10-fold volume of 95% ethanol with continuous stirring at room temperature for 16 h. The ethanol extracts were dried by evaporation under reduced pressure in a rotary evaporator and were dissolved in DMSO to a final concentration of 37 mg/ml. For preliminary screening, ethanol extracts from whole plants of 101 different species from 75 families collected from Taiwan were tested at a concentration of 10 μg/ml in 1% DMSO. The ethanol extracts of the second batch of Hit 4 (*A. spinosus*) and two closely related species, *A. viridis* (Hit 4-1) and *A. tricolor* (Hit 4- 2) were prepared using the same method. For further fractionation, the ethanol extracts were condensed and stored at 4°C for further fractionation. Large-scale preparations of *A. viridis* extracts were prepared by the Industrial Technology Research Institute, Hsinchu, Taiwan. Briefly, fresh *A. viridis* plants (500 Kg) were collected, washed with water, dried in an oven at 65°C for 13 h, and chopped into small strips. The dried strips (47.3 kg) were extracted by 95% ethanol reflux extraction for 24 h with intermittent stirring at 2 h intervals, repeated once. The extract was concentrated via an evaporator to a final solid content of 6.43% in 25.7 L of ethanol. The extract was stored at 4°C before use.

Partition and fractionation of the ethanol extract of Amaranthus viridis L.

The ethanol extract was further fractionated into different fractions as illustrated in Figure S2. The ethanol extract was loaded onto a Diaion HP-20 column (80*240 mm) and eluted with a stepwise sequence of 100% H_2O (F1), 50% ethanol EtOH/H_2O (v/v) (F2), 100% EtOH (F3), 50% EtOH/ethanol-ethyl acetate (EtOAc) (v/v) (F4), 100% EtOAc (F5). Each fraction was concentrated by an evaporator and stored at -20°C until further use. The F4 fraction obtained from the large-scale *A. viridis* preparation was further fractionated by phase chromatography on silica gel into 107 fractions.

Analytical thin layer chromatography (TLC) and pooling of fractions

The content of each eluted fraction was spotted on pre-coated (silica gel F254) aluminium plates in a small chromatographic tank. Fractions were characterized based on their relative mobilities in solvent systems and color reactions with UV light. The fractions were pooled into 12 fractions based on the similarity of the TLC patterns (F4-IS1 to F4-IS12).

Identification of bioactive compounds from A. viridis that inhibit FGFR3 activation

The most potent active fractions to inhibit FGFR3 activity in the cell-based assay system were further fractionated and the main chemical constituents of active fractions were identified. Each fraction was tested for cytotoxicity and its ability to inhibit FGFR3 activity using the cell-based assay system. The most potent fraction without cytotoxicity was further fractionated, and the main chemical constituents were identified by AnlytiCon, Potsdam, Germany. F4-IS11 and F4-IS-12 fractions were further fractionated into 227 fractions (Supplemental Figure 3), and finally 8 fractions (Supplemental Figure 4). The final 2 most potent fractions (N100-N3 and N100-N5) showed high purity based on HPLC and MS analysis (Supplemental Figure 4) were analyzed for their main chemical constituents using high-resolution MA, MS/MS, IR and NMR.

Pa structurally related molecules

Pheophorbide a (# 16072), Temoporfin (#17333) and 5-Aminolevulinic Acid (hydrochloride) (#9001902) were purchased from Cayman chemical. Pyropheophorbide-a (# sc-264178), chlorophyllin sodium copper salt (# sc-227637), and pyrophenophorbide-a methyl ester (# sc-26409) were purchased from Santa Cruz Animal Health.

Primary chondrocytes isolation

Murine articular chondrocytes were harvested as previously described (1). Briefly, the articular cartilage from homozygous *FGFR3*^{ACH} mice was isolated on postnatal day 5, followed by digestion with 3 mg/ml collagenase D (Roche) in DMEM culture medium for 45 minutes at 37°C and continued digestion with diluted digestion solution (0.5 mg/ml collagenase D) at 37°C overnight. The suspension of isolated cells was filtered through a 48 μm cell strainer for subsequent counting and seeding.

RNA isolation and quantitative real-time RT-PCR analysis

Total RNA from various cell lines was isolated using TRIzol reagent (Invitrogen). Next, total RNA was further purified with the QIAGEN RNeasy Mini Kit, treated with DNase (DNase I, 30 U/μg total RNA; QIAGEN), and reverse transcribed using the SuperScript III First-Strand Synthesis System (Invitrogen). The levels of FGFR1, 2, 3 and 4 mRNA were quantitatively detected by real-time RT-PCR using SYBR Green PCR Master Mix and an ABI Prism 7900HT Sequence Detection System (Applied Biosystems). The primers used for RT-PCR are as follows:

FGFR1

Forward: 5′-GAGATGGAGGTGCTTCACTTA-3′

Reverse: 5′-TACAGGGGCGAGGTCATCA-3′

FGFR2

Forward: 5′-ATGCTTGTACTGCCAGTAGGACTGT-3′

Reverse: 5′-CTGACAAAATCTTCCGCACCAT-3′

FGFR3

Forward: 5′-CCTCGGGAGATGACGAAGC-3′

Reverse: 5′-CGGGCCGTGTCCAGTAAGG-3′

FGFR4

Forward: 5′-TGCAGAATCTCACCTTGATTACA-3′

Reverse: 5′-GGGGTAACTGTGCCTATTCG-3′

GAPDH

Forward: 5′-TTCGCTCTCTGCTCCTCCTGT-3′

Reverse: 5′-GCCCAATACGACCAAATCCG-3′

Gapdh

Forward: 5′-CCATGGAGAAGGCTGGGG-3′

Reverse: 5′-CAAAGTTGTCATGGATGACC-3′

Denaturing immunoprecipitation

Cells were treated with vehicle or 20 μM Pa for 30 minutes in supplemented with 30 μg/ml heparin and 10 ng/ml FGF2. Then, denaturing FGFR3 immunoprecipitation was performed as described previously (2). Briefly, protein extracts were prepared using denaturing lysis buffer (0.6 % SDS, 5 mM Tris-HCl, 5 mM EDTA, 5 mM EGTA, 10 μM iodoacetamide, and 1x Protease and Phosphatase inhibitor Cocktail). The lysates were heated at 100°C for 3 minutes following passed through the QIAshredder (QIAGEN), and further dilution with 2.5 volumes of immunoprecipitation dilution buffer (1.2% Triton X-100, 100 mM NaCl, 20 mM sodium borate, 15 mM EDTA, 15 mM EGTA, 0.7% BSA, 10 μM iodoacetamide and 1x Protease and Phosphatase Inhibitor Cocktail). FGFR3 was immunoprecipitated using FGFR3 antibody (Cell Signaling, #4574) and Protein A Mag Sepharose beads (Cytiva, 28-9670-62). Protein samples were subjected to SDS-PAGE and transferred to PVDF membranes. Western blot analysis was performed using ubiquitin (P4D1) antibody (Cell Signaling, #3936) and FGFR3 antibody. Signal detection was performed using ECL, and images were acquired using UVP Biospectrum 600 imaging system.

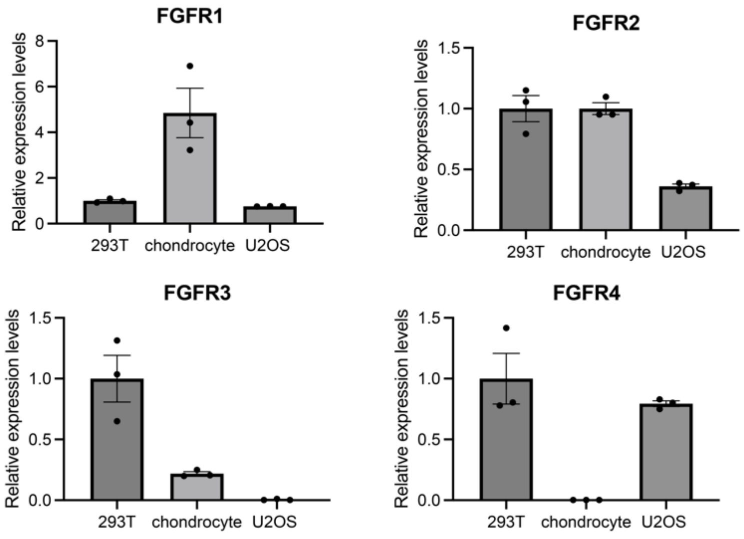

Supplemental Figure S1. Relative expression levels of FGFR1-4 mRNA in three different cell lines. FGFR1, 2, 3, and 4 mRNA levels in U2OS cells, 293T cells, and chondrocytes. Human adult articular chondrocytes were obtained from surgical waste following total knee replacement surgery. Expression of FGFR1, 2, and 4 was detected in U2OS cell; however, FGFR3 expression was not detected.

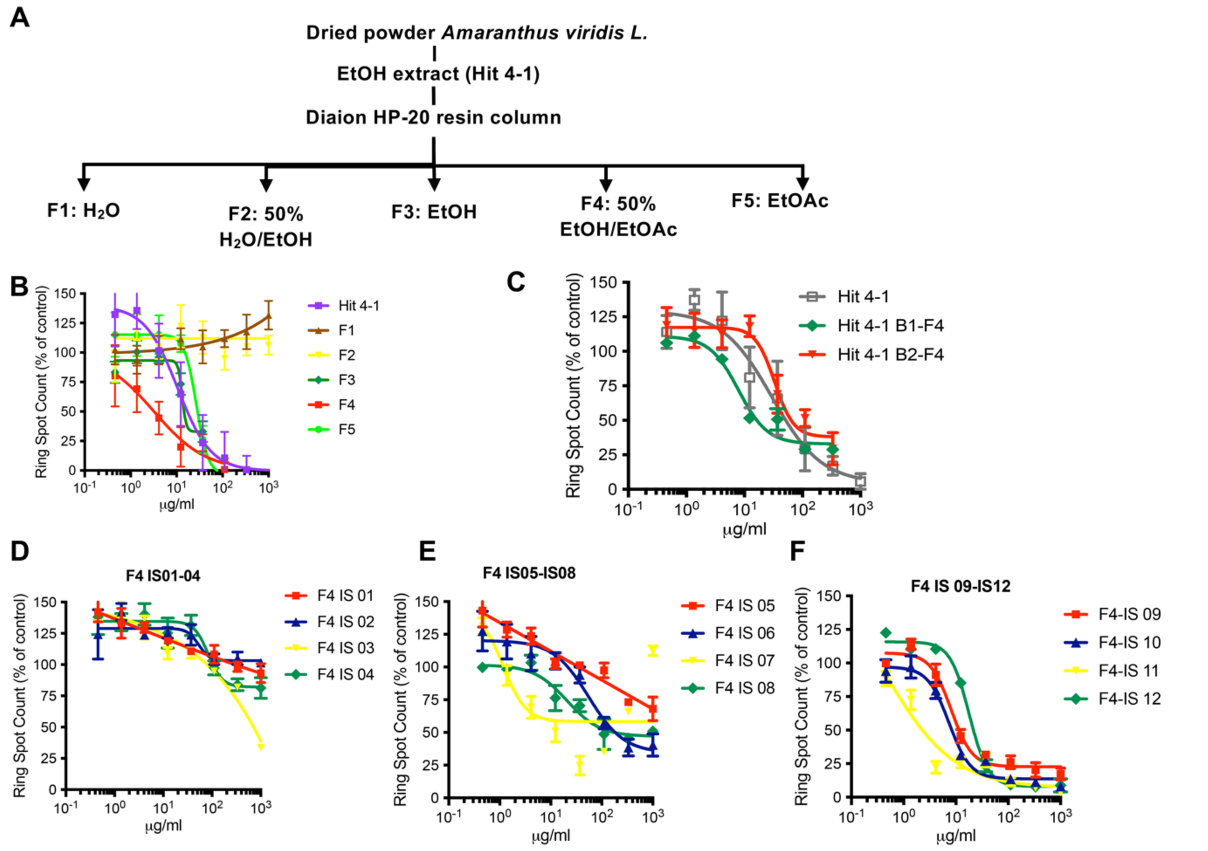

Supplemental Figure 2. Inhibition of FGFR3 activation by sequential fractions of *A. viridis.* extracts. (**A**) Flow chart showing the fractionation scheme as described in the Material and Methods section. (**B-F**) Quantification of FGFR3 activity using R/ATA system. The dose-response curves revealed the FGFR3 inhibitory activities of (**B**) five partitioned fractions from extracts of *A. viridis*, (**C**) F4 fractions from two different preparations indicate batch-to-batch consistency, (**D-F**) 12 fractions derived from fraction F4. **B-F**, Data are shown as mean ± SEM of triplicates.

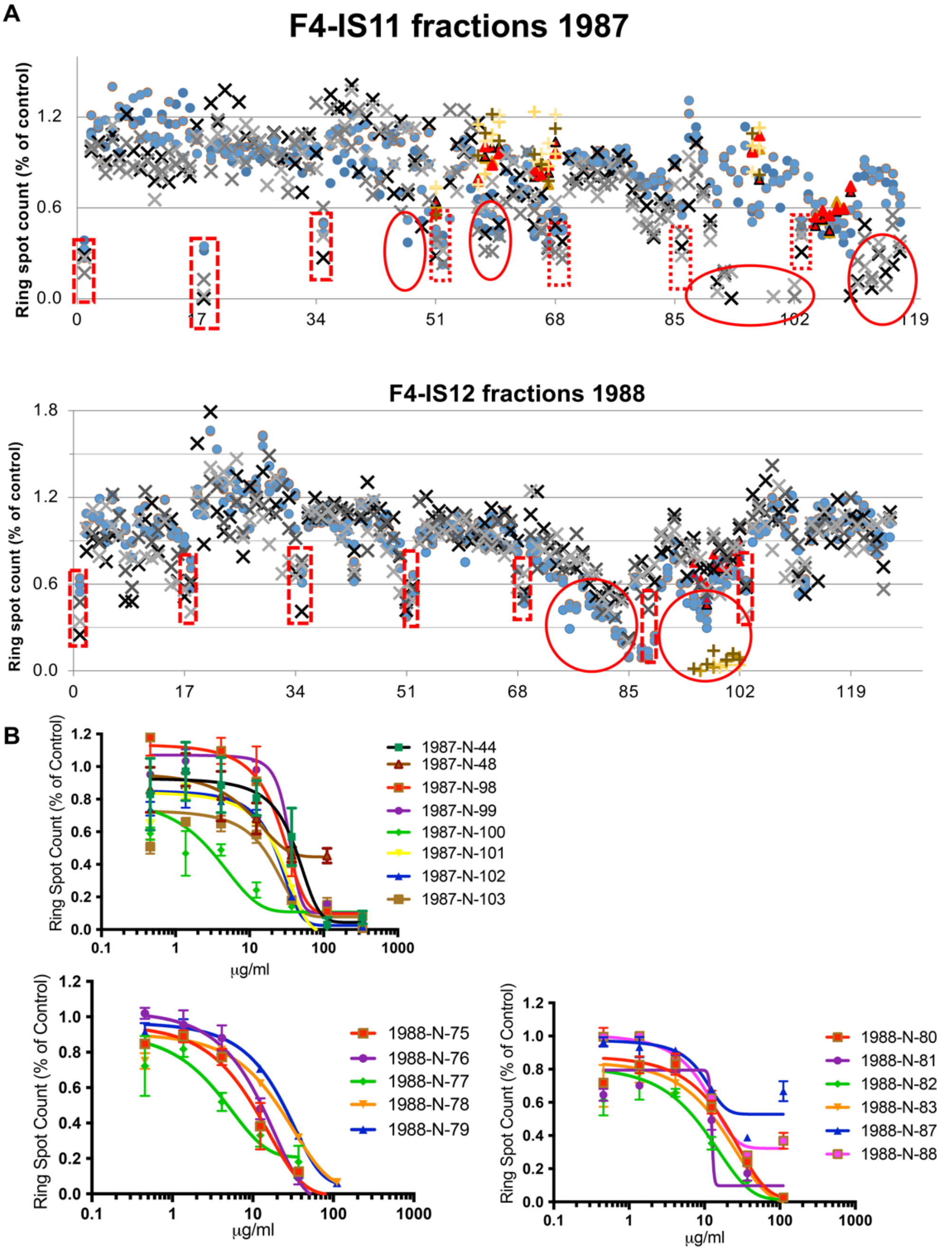

Supplemental Figure 3. Inhibition of FGFR3 activation by sequential fractions of F4-IS11 and F4-IS12. (**A**) FGFR3 activity assay results from sub-fractions of the IS11 and IS12 fractions using R/ATA system, based on five to seven independent experiments from each sub-fraction. Rectangles with broken lines indicate PKC412 treatment controls. The most potent fractions (circles with solid line), producing ring spot counts per cell less than 60% of DMSO controls (mean ±SEM), were selected for subsequent dose-response assays. **(B)** Dose-response curves from the sub-fractions selected from (**A**). Data are shown as mean ± SEM of triplicates.

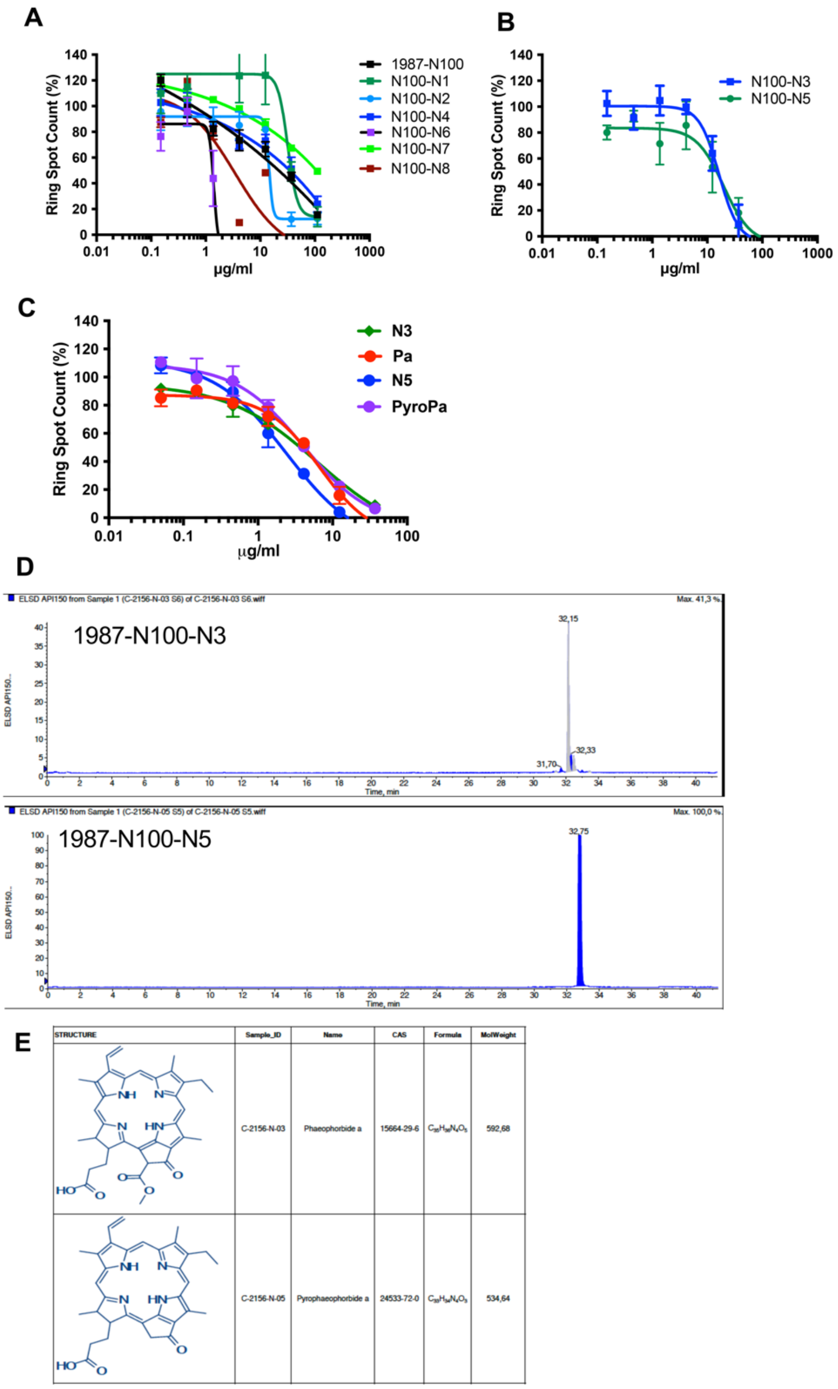

Supplemental Figure 4. Inhibition of FGFR3 activation by sub-fractions of 1987-N100 and purified compounds, Pa and PyroPa. (**A-C**) Dose-response curves using R/ATA system. (**A-B**) sub-fractions from the 1987-N100 fraction. (**C**) Pa and PyroPa inhibited FGFR3 activities in compared with 1987-N100-N3 and N5. **A-C**, Data are shown as mean ± SEM of triplicates. (**D**) Sub-fraction, 1987-N100-N3 and 1987-N100-N5 were analyzed using HPLC in combination with MS/NMR. (**E**) The molecular structure and molecular weight of identified compounds, Pa and PyroPa.

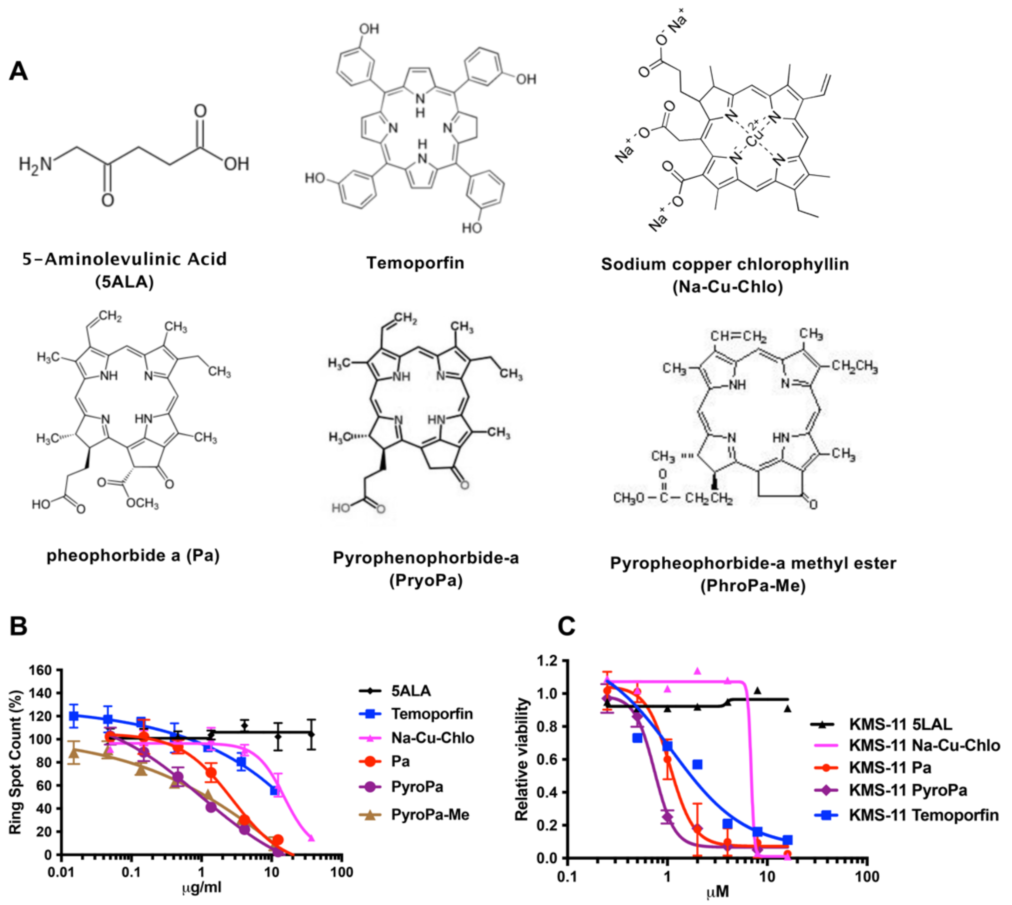

Supplemental Figure 5. Pa, PyroPa, and structurally related molecules inhibited FGFR3 activity. (A) Structures of several porphyrin derivatives, including Pa, PyroPa, temoporfin, chlorophyllin A (Na-Cu-Chlo), pyropheophorbide-a methyl ester (PhroPa-Me), and a porphyrins precursor, 5-aminolevulinic acid (5ALA). (B) Dose-response of compounds in terms of FGFR3 inhibition. (C) Compounds shown to inhibit viability of KMS-11 cells. Relative cell viability was normalized to the mock-treated control. KMS-11 and U2OS cells were treated with indicated doses of the compounds for 48 h, and cell viability was analyzed by WST-1. Data are shown as mean ± SEM from three independent experiments.

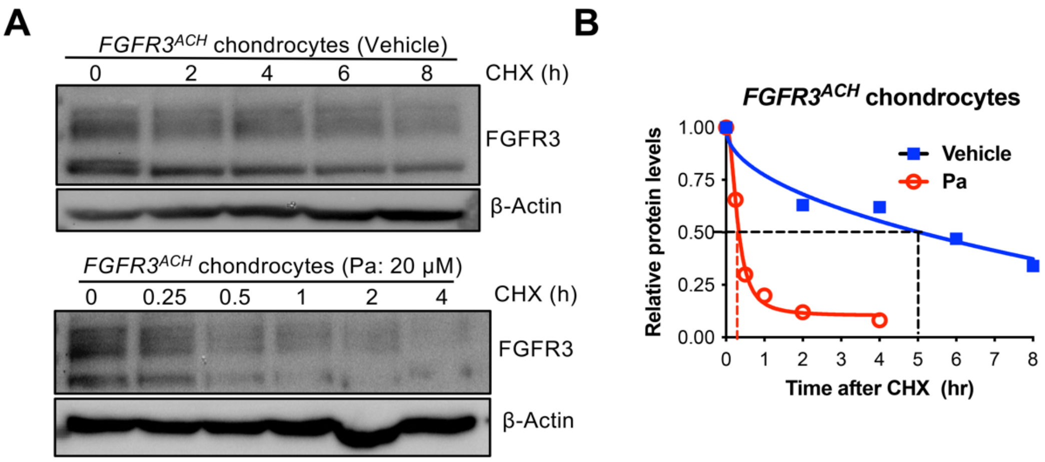

Supplemental Figure 6. Pa compromises FGFR3 protein stability in primary chondrocytes isolated from homozygous *FGFR3*^{ACH} mice. *FGFR3*^{ACH} chondrocytes were treated with vehicle or 20 μM Pa in the presence of 100 μg/mL CHX for the indicated times. (A) Protein levels were detected via immunoblotting. (B) The relative FGFR3 protein levels were quantified, normalized to β-actin, and compared to time 0 of CHX treatment.

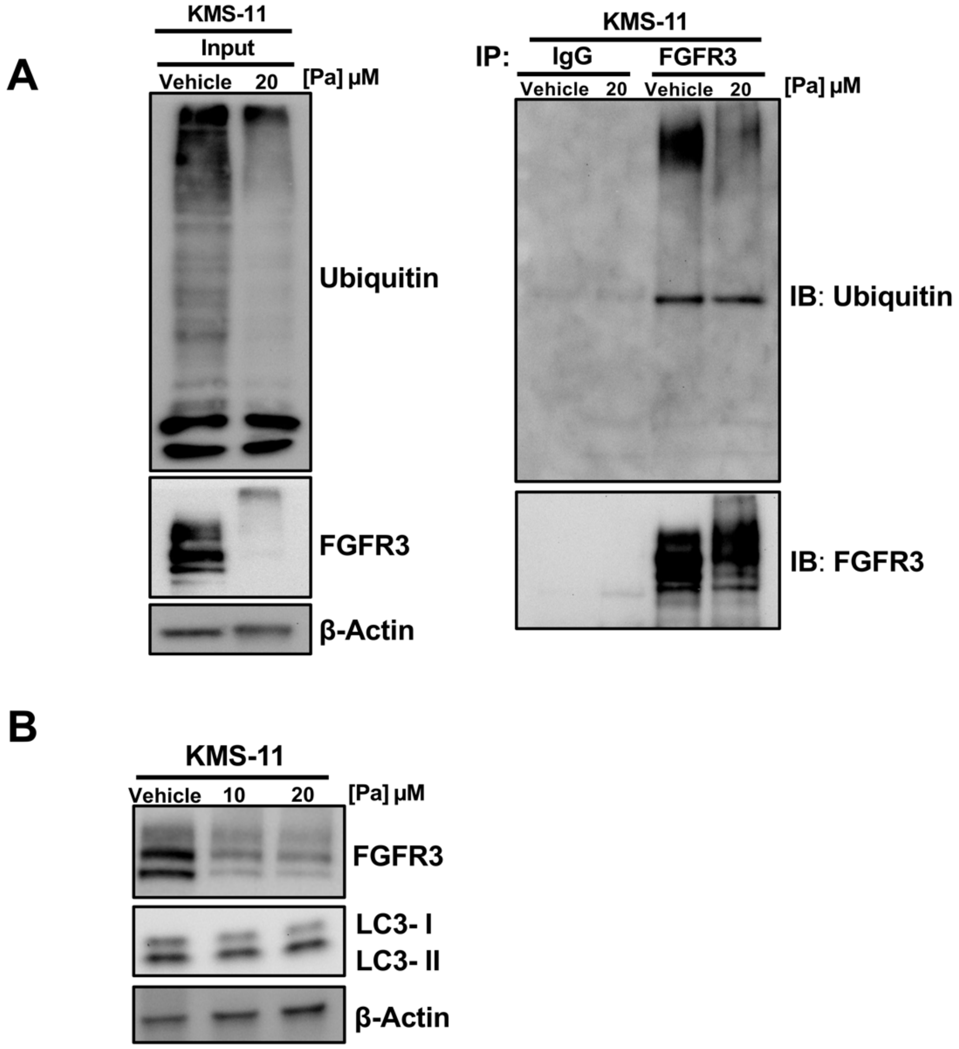

Supplemental Figure 7. Pa-induced FGFR3 degradation is not mediated by either ubiquitination or autophagy. (**A**) KMS-11 cells were preincubated with bafilomycin A1 (100 nM) for 4 hours to inhibit the protein lysosomal degradation before vehicle or Pa treatment in the presence of heparin (30 μg/ml) and FGF2 (10 ng/ml) for 30 minutes. Cell lysates were immunoprecipitated (IP) of FGFR3 under denaturing conditions and immunoblotted with FGFR3 or ubiquitin antibodies. (**B**) KMS-11 cells were treated with vehicle or Pa at the indicated concentration for one hour in the presence of ligand stimulation. Protein levels of LC3-I and LC3-II were determined by Western blot analysis.

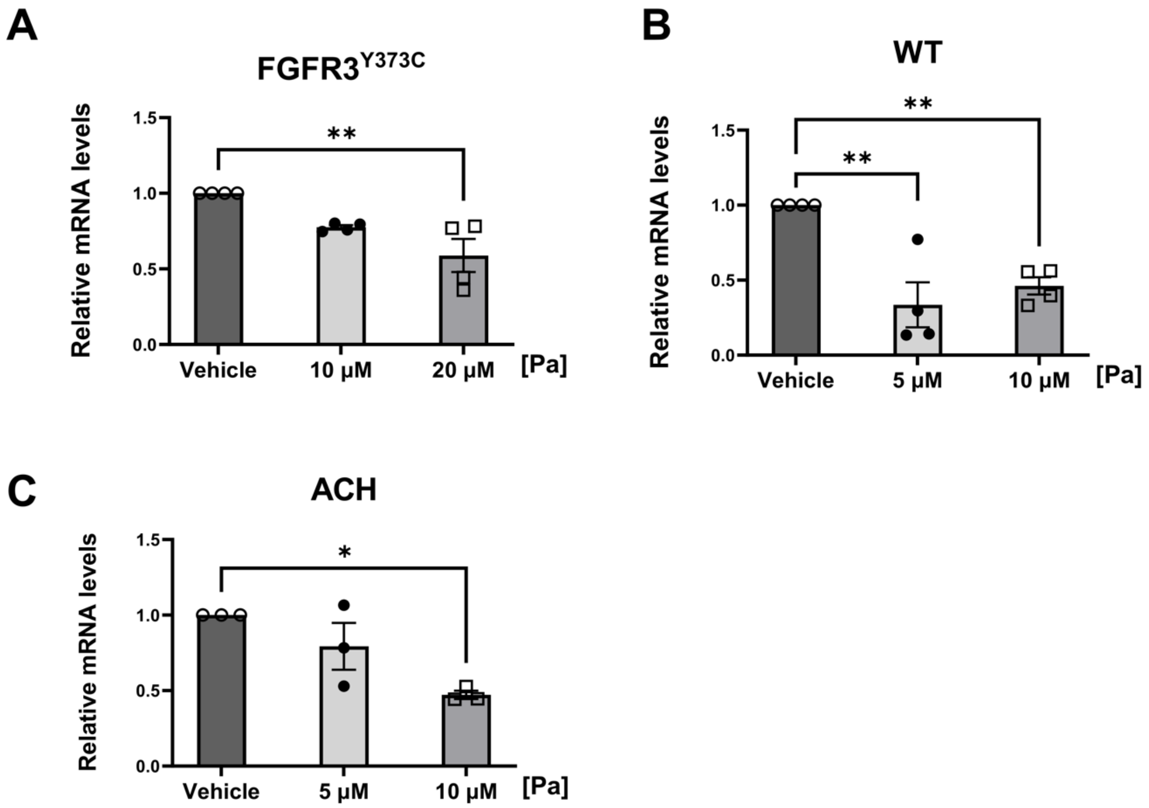

Supplemental Figure 8. Transcriptional repression of FGFR3 by Pa. KMS-11 (**A**), WT (**B**) and ACH FGFR3-expressing (**C**) ATDC5 cells were treated with vehicle or Pa as indicated concentration for one or two hours in the presence of heparin (30 µg/ml) and FGF2 (10 ng/ml) or FGF2 (20 ng/ml). The expression levels of FGFR3 mRNA were determined by real-time PCR. Data were presented in relative gene expressions normalization using GAPDH and compared to their vehicle treated controls. Data are shown as mean ± SEM from three to four independent experiments. Statistical analysis was performed using one-way ANOVA. (* p-values less than 0.05: ** p-values less than 0.01)

References

1. Gosset M, Berenbaum F, Thirion S, and Jacques C. Primary culture and phenotyping of murine chondrocytes. *Nature protocols.* 2008;3(8):1253-60.
2. Laederich MB, Degnin CR, Lunstrum GP, Holden P, and Horton WA. Fibroblast growth factor receptor 3 (FGFR3) is a strong heat shock protein 90 (Hsp90) client: implications for therapeutic manipulation. *J Biol Chem.* 2011;286(22):19597-604.
